# Tuo-Min-Ding-Chuan Decoction Alleviate Ovalbumin-Induced Allergic Asthma by Inhibiting Mast Cell Degranulation and Down-Regulating the Differential Expression Proteins

**DOI:** 10.3389/fphar.2021.725953

**Published:** 2021-09-22

**Authors:** Jingbo Qin, Mingsheng Lv, Zeqiang Jiang, Xianghe Meng, Yi Wang, Jiarui Cui, Ji Wang, Qi Wang

**Affiliations:** ^1^ School of Traditional Chinese Medicine, Beijing University of Chinese Medicine (BUCM), Beijing, China; ^2^ Respiratory Department, BUCM Third Affiliated Hospital, Beijing, China; ^3^ National Institute of TCM Constitution and Preventive Medicine, BUCM, Beijing, China

**Keywords:** TMDCD, allergic asthma, Chinese herbal compound, mast cells, differential expression proteins

## Abstract

Allergic asthma is a stubborn chronic inflammatory disease, and is considered a co-result of various immune cells, especially mast cells, eosinophils and T lymphocytes. At present, the treatment methods of allergic asthma are limited and the side effects are obvious. Traditional Chinese medicine has been used to treat diseases for thousands of years in China. One such example is the treatment of allergic asthma, which take the characteristics of less adverse reactions and obvious curative effect. Tuo-Min-Ding-Chuan Decoction (TMDCD) is a traditional Chinese medicine compound for the treatment of allergic asthma optimized from Ma-Xing-Gan-Shi Decoction (MXGSD), which was put forward in Treatise on Febrile Diseases by Zhang Zhongjing in the Eastern Han Dynasty. The compound shows a significant clinical effect, but the mechanism of its influence on the immune system is still unclear. The purpose of this study was to observe whether TMDCD could alleviate the symptoms of ovalbumin (OVA) challenged allergic asthma mice, and to explore its immune regulatory mechanism, especially on mast cell (MC) degranulation. The results showed TMDCD could not only reduce the airway hyperresponsiveness (AHR), inflammatory cell infiltration and mucus secretion in the lung tissue of OVA challenged mice, but also decrease the levels of total IgE, OVA-specific IgE, histamine and LTC4 in serum. We found that TMDCD can downregulate the expression of Fractalkine, Tryptase ε, IL-25, CCL19, MCP-1, OX40L, Axl, CCL22, CD30, G-CSF, E-selectin, OPN, CCL5, P-selectin, Gas6, TSLP in OVA challenged mice serum by using mouse cytokines antibody array. It has been reported in some literatures that these differentially expressed proteins are related to the occurrence of allergic asthma, such as tryptase ε, MCP-1, CCL5, etc. can be released by MC. And the results of *in vitro* experiments showed that TMDCD inhibited the degranulation of RBL-2H3 cells stimulated by DNP-IgE/BSA. Taken together, we made the conclusion that TMDCD could reduce the infiltration of inflammatory cells in lung tissue and alleviate airway remodeling in mice with allergic asthma, showed the effects of anti-inflammatory and antiasthmatic. TMDCD could also reduce the levels of IgE, histamine, LTC4, Tryptase ε, and other MC related proteins in the serum of allergic asthma mice, and the *in vitro* experiments showed that TMDCD could inhibit IgE mediated degranulation and histamine release of RBL-2H3 cells, proved its anti allergic effect.

## Introduction

Allergic asthma is a chronic inflammatory disease, which is known as the result of hypersensitivity to inhaled antigens and is driven by aberrant innate and adaptive immune responses, the cardinal features include mucus hypersecretion, pulmonary eosinophilic infiltration, and AHR (Thio et al., 2018). According to the (The Global Asthma Report 2018, 2018), over 339 million people worldwide suffer from asthma (E, 2018). Currently, asthma occurs in any countries, and over 80% of asthma cases occur in low- and lower-middle income countries. The increase of asthma patients is associated with increasing air pollution by rapid urbanization and industrialization, and adds a serious burden on the health system and patients’ quality of life (Dhar et al., 2020). At present, corticosteroids were considered as the most effective treatment in asthma, for they may effectively control symptoms and prevent asthma exacerbation (Ora et al., 2020). The 2019 Global Initiative for Asthma (GINA) guidelines suggested adult asthma patients be treated with corticosteroids inhaling daily, or as needed, and reduced asthma triggers as much as possible (Mauer and Taliercio, 2020). However, the clinical safety of long-term intaking corticosteroids is still controversial. Some researchers have found that long-term or repeated short-term use of corticosteroids may lead to certain side effects on systemic health (Price et al., 2018; Matsunaga et al., 2020). Therefore, it’s an urgent problem for researchers to find some safe and effective drugs for asthma, alternatively.

MC undertakes important roles in the development of allergic asthma. Clinical study has shown that the level of lung function in young adults with allergic asthma is related to blood circulating mast cell progenitors (MCPs) (Salomonsson et al., 2019). MCPs migrate from the blood into the airway, exposed to some key factors, which could induce them to “mature,” then finally transferred into a pivotal immunomodulatory cell (Penn, 2020). Different biological inducers (e.g. SCF, CCL5, CXCL8, and CXCL10, etc.) are responsible for recruiting MC to airway epithelium and airway smooth muscle (ASM) (Elieh Ali Komi et al., 2019). The cross-linking of MC binding IgE by allergens leads to the release of biologically active mediators (e.g. histamine, PGD2, LTC4) through degranulation, which promotes ASM contraction, mucosal edema and mucus secretion in allergic asthma directly (Penn, 2020). Moreover, the activated MC secrete a variety of cytokines, leading to anaphylactic reaction through recruitment and activation of eosinophils, neutrocyte, and Th2 cells, together with the interaction between MC and histocytes in the lesion site (Mukai et al., 2018). Thus, MC is considered as a key driver of long-term pathophysiological changes and tissue remodeling, which are related to chronic allergic inflammation in asthma (Galli and Tsai, 2012).

Antihistamines and steroids are effective drugs in the treatment of allergic diseases, but serious side effects hinder their long-term use (Lim et al., 2020). TMDCD is a Chinese herbal compound optimized based on the compound of MXGSD and GuoMinKang, and also is a summary of clinical experience on allergic asthma by professor Wang Qi from Beijing university of Chinese medicine. The MXGSD, recorded in Treatise on Febrile Diseases by Zhang Zhongjing in Han Dynasty, was used to dispersing lung and relieving asthma. Some researchers have found that MXGSD could alleviate inflammatory reaction and reduce airway remodeling in lung tissue of asthma animal model (Yu et al., 2017; Song et al., 2018). MXGSD was also shown the benefit in improving lung function and the level of fractional exhaled nitric oxide (FeNO) (Ma and Gong, 2019; Cai et al., 2020). Our previous studies have shown that Guo-Min-Kang could decrease the serum specific IgE, IL-4, IL-5 and IL-13 levels in patients with allergic diseases, and improve the tolerance of the human body to antigen (Bao, 2017). TMDCD can ameliorate asthma symptoms and reduce the frequency of asthma attacks clinically, but the mechanism is not clear. Based on the above situation, this study focuses on the immunomodulatory effect of TMDCD on allergic asthma and mast cells (RBL-2H3) in mice with allergic asthma, to elucidate the mechanism of TMDCD in allergic asthma.

## Materials and Methods

### Reagents and Instruments

In this study, we used the following reagents: Acetonitrile and Alum were purchased from Thermo Fisher (USA); while Formic acid (HPLC grade), albumin (OVA), Dexamethasone (for *in vitro* experiments), Methacholine (MeCh), 0.25% Trypsin-EDTA, 4-Nitrophenyl N-acetyl-β-D-glucosaminide, and mouse monoclonal anti-Dinitrophenyl antibody (DNP-IgE) from Sigma (USA); Glycogen periodic acid schiff (PAS) stain Kit, Penicillin-Streptomycin liquid, Triton X-100, Toluidine Blue O solution from Solarbio (China). Dexamethasone (for *in vivo* experiments) from Lisheng-Pharma (China); Mouse IgE ELISA Kit and Mouse OVA sIgE ELISA Kit from CUSABIO (China); Histamine ELISA Kit and Mouse LTC4 ELISA Kit from CLOUD-CLONE (China); the Mouse cytokine antibody array (QAM-CAA-4000) from RayBiotech (China). The FBS, MEM medium, and GlutaMAX were purchased from Gibco (USA), DMSO (MPBIO, China), PIPES Buffer solution (pH 7.2) and 0.1 M carbonate buffer from Coolaber (China), DNP-conjugated bovine serum albumin (DNP-BSA, Biosearch, China), CCK-8 Kit (Dojindo, Japan) were prepared, respectively.

The following instruments were employed: Ultra performance liquid chromatography (UPLC, Shimadzu, Japan), Votex and CO_2_ incubator (Thermo, USA), Mass spectrometer (AB SCIEX, USA), Electronic balance (Shimadzu, Japan), Atomizer (OMRON, Japan), Unconstrained whole-body plethysmography system (WBPs, DSI, USA), Resistance and Compliance Plethysmographs (RCs, DSI, USA), high-speed benchtop refrigerated centrifuge (Microfuge22R, Beckman Coulter, Germany), Blood cell analyzer (NIHON KOHDEN, Japan), Microtome (Leica, Germany), Enzyme-labeled instrument (BioTeK, USA), Fluorescence scanner (InnoScan, France).

### TMDCD Preparation

TMDCD is composed of 12 kinds of traditional Chinese medicine (Table 1), the ingredients were purchased from Beijing Tong Ren Tang Technology Development Co., Ltd. (Beijing, China), and were certified as a qualified product. *Prunus mume* (Siebold) Siebold and Zucc (batch No.190728004), *Cicadae Periostracum* (batch No. 900002134), *Reynoutria multiflora* (Thunb.) Moldenke (batch No. 90706003), *Ganoderma lucidum* (Leyss. ex Fr.) Karst. (batch No.001001212A), *Saposhnikovia divaricata* (Turcz. ex Ledeb.) Schischk (batch No.90917001), *Gastrodia elata Blume* (Orchidaceae) (batch No.20041302), *Ephedra sinica Stapf* (batch No.18090402), *Prunus armeniaca* L. (batch No.900021975), *Gypsum Fibrosum* (batch No.190724001), *Glycyrrhiza uralensis* Fisch. ex DC. (batch No.200352), *Bombyx mori* L. (batch No.180642), *Fagopyrum cymosum* (Trevir.) Meisn. (batch No.901001876). The preparation method of TMDCD was as follow: 1) A total of 780 g of the 12 medicinal materials were weighed, respectively, according to the proportion of TMDCD in Table 1, then they were soaked together in 7800 ml (10 times the weight of medicinal materials) deionized water for 30 min, followed a 100 min of decoction under 100°C, and the extracting solution was collected. 2) Added 7800 ml deionized water again, the same decocting conditions, and collected the solution. 3) Repeat the second step once. Finally, all the collected solution was concentrated to 770 ml (the relative density of 1.01 g/ml, equals to 20.2 g/kg/d). More details can be found in references (Wu et al., 2019). The voucher for raw material specimens was deposited in the principal investigator’s laboratory (Supplementary Figure S1).

**TABLE 1:** TMDCD composition.

Component (*Latin*)	Weight (g)
*Prunus mume* (Siebold) Siebold & Zucc (*Fructus Mume*)	20
*Cicadae Periostracum (Pharmacopoeia of China (2015)) (Periostracum Cicadae)*	10
*Reynoutria multiflora* (Thunb.) Moldenke (*Caulis Polygoni Multiflori*)	15
*Ganoderma lucidum* (Leyss. ex Fr.) Karst. (*Ganoderma*)	10
*Saposhnikovia divaricata* (Turcz. ex Ledeb.) Schischk (*Radix Saposhnikoviae*)	10
*Gastrodia elata* Blume (Orchidaceae) (*Rhizoma Gastrodiae*)	10
*Ephedra sinica* Stapf (*Herba Ephedrae*)	10
*Prunus armeniaca* L. (*Semen Armeniacae Amarum*)	10
*Gypsum Fibrosum (Pharmacopoeia of China (2015)) (Gypsum Fibrosum)*	30
*Glycyrrhiza glabra* L. (*Radix Glycyrrhizae*)	6
*Bombyx mori* L. (*Bombyx Batryticatus*)	10
*Fagopyrum cymosum* (Trevir.) Meisn. (*Rhizoma Fagopyri Dibotryis*)	15

### LC-MS/MS

The TMDCD extract was centrifuged by high-speed benchtop refrigerated centrifuge at 12,000 rpm for 10 min, and the supernatant was blended by vortexing, and filtered through a 0.45 μm micropore membrane prior to injection. The sample was stored at 4°C before analysis.

Chromatographic analysis was performed on UPLC LC-30A System equipped with an online degasser, a binary pump and an autosampler. Chromatographic separation was performed on a SHIMADZU InerSustain C18 column (100 × 2.1 mm, 2 µm). The composition of the mobile phase is A.Equate = “acetonitrile”, B.Equate = “0.1% HCOOH-H_2_O”. The column temperature was 35°C. The flow rate was 0.3 ml/min. The injected sample volume was 5 µl. (See chromatographic conditions in Supplementary Table S1).

The two modes of positive and negative ions in electrospray ionization (ESI) were used to detect the detected substance. The ESI source conditions are as follows: Ion Source Gas1 (Gas 1):50, Ion Source Gas2 (Gas 2):50, Curtain Gas (CUR): 25, Source Temperature: 500°C (positive) and 450°C (negative), Ion Sapary Voltage Floating (ISVF) 5500 V (positive) and 4400 V (negative), TOF MS scan range: 100–1200 Da, production scan range: 50–1000 Da, TOF MS scan accumulation time 0.2 s, production scan accumulation time 0.01 s. The secondary mass spectrometry was obtained by information-dependent acquisition (IDA), high sensitivity model was used. Declustering potential (DP): ±60 V, Collision Energy: 35 ± 15 eV.

Import the original data of LC-MS into MS-DIAL (data-independent MS/MS deconvolution for comprehensive metabolome analysis) V4.20 for pre-processing, including peak extraction, denoising, deconvolution, and peak alignment. A three-dimensional data matrix (original data matrix) in CSV format containing sample information, retention time, mass nucleus ratio and mass spectrum response intensity (peak area) was deriving. Then the information was searched with three databases: MassBank, Respect, GNPS (a total of 14,951 records).

### Animals

The experimental animals were all provided by Beijing Vital River Laboratory Animal Technology Co., Ltd. (Beijing, China). Female BALB/c mice aged 7–8 weeks (18–20 g) eat and drink freely were used (Animal Batch No: SCXK (Beijing) 2016-0006), and were reared under specific pathogen-free (SPF) conditions. The experimental protocols were approved by the Animal Research Ethics Committee of Beijing University of Chinese Medicine. We included 12 mice for each group, of which six were used for the detection of WBPs and other indicators in the study, the other six were used for RCs detection. It should be noted that the detection process of WBPs and RCs may lead to the death of animals, if so, the results of the death sample will not be used. Therefore, the number of mice in each group varied in the results section.

### Establishment of Allergic Asthma Model and Drug Treatment

**FIGURE 1:**
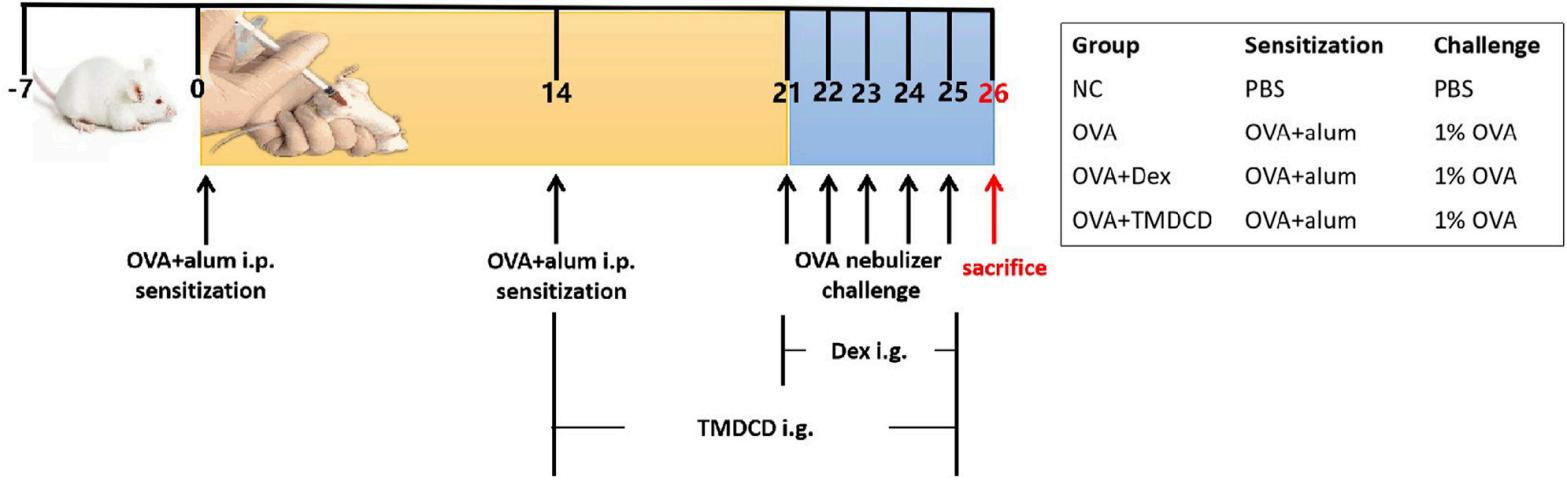
Schematic diagram of allergic asthma model establishment. Sensitization: The mixture of OVA and aluminum hydroxide gel was given to BALB/c mice (i.p.) at day 0 and day 14. Challenge: 1% OVA was given by aerosol inhalation to mice in all the groups except NC group (given PBS, instead) from day 21 to day 25, continuously. Treatment: The TMDCD group mice were given TMDCD by gavage after day 14, altogether for 12 days. From day 21 to day 25, 1 h before OVA challenge, gave dexamethasone to the mice in OVA + Dex group by gavage. The mice were sacrificed on day 26.

The mice asthma model was established according to the reference method (Reddy et al., 2012). Given 0.2 ml/mice sensitizer (Mixture of OVA and aluminum hydroxide gel) to the mice in allergic asthma group (OVA), Dexamethasone group (OVA + Dex), and TMDCD group by intraperitoneal injection (i.p.), while 0.2 ml/mice PBS to PBS group (NC), at day 0 and day 14.1% OVA was given to OVA group, OVA + Dex group and OVA + TMDCD group by aerosol inhalation for 30 min, respectively, on days 21, 22, 23, 24 and 25, to challenge asthma. PBS, instead of 1% OVA was given to the NC group as a control. Synchronously with the above process, the mice in the OVA + TMDCD group were given TMDCD (20.2 g/kg/d) (The dosage is determined according to the results of pre-experiment, which is not described in this paper.) by gavage started from day 14, continuously, to day 25, while PBS was given to mice in other groups. It should be noted that the OVA + Dex group was changed to give dexamethasone (0.5 mg/kg/d) by gavage at day 21, 5 days in all, to day 25. The sensitization, challenge, and dosing scheme are shown in Figure 1.

### Airway Hyperresponsiveness (AHR)

When the last nebulization for asthma challenging finished 24 h, move the mice into the atomization chamber of WBPs in an awakened state. Setting system parameters, acclimation time: 5 min, aerosol volume: 2 ml, nebulization time: 2 min, response time: 3 min, recovery time: 1 min. The nebulization concentration of MeCh were 0 mg/ml, 3.125 mg/ml, 6.25 mg/ml, 12.5 mg/ml, 25 mg/ml, from low to high, respectively. The Penh value and the Pause value were used to reflect the degree of bronchial contraction.

Synchronously, the mice were anesthetized with 1% Pentobarbital, followed by tracheal intubation, then move the mice into the atomization chamber, the endotracheal tube was connected to the RCs. Setting system parameters, acclimation time: 5 min, nebulization time: 30 s, aerosol volume: 20 μl, response time: 3 min, recovery time: 3 min. Setting ventilator parameters, Max stroke volume: 0.2 ml, Max mouth pressure: 30 cm H_2_O, Deep inflation max volume: 0.75 ml, Deep inflation max pressure: 40 cm H_2_O, Rate: 140 breaths/min, PEEP: 0 cm H_2_O. The nebulization concentration of MeCh were 0 mg/ml, 3.125 mg/ml, 6.25, 12.5, 25 mg/ml, from low to high, respectively. Record R_L_ and Cdyn values.

### Collection of Bronchoalveolar Lavage Fluid (BALF)

The mice were anesthetized with 1% Pentobarbital, tracheal intubation was performed after blood collection. Pour 1 ml cold PBS into the mice lung, about 0.8 ml of bronchoalveolar lavage fluid (BALF) was recycled. The supernatant separated by centrifugation was stored at −80°C, and resuspend the precipitate by PBS, the cells were classified and counted by Blood cell analyzer.

### Lung Histopathology

Fixed mice lung tissue with 4% Paraformaldehyde, and embedded in paraffin. Tissue sections (4 μm) were made, and dyed with H&E and PAS staining. Examined the inflammation and mucus secretion around the bronchi and blood vessels of the lung under the microscope, and photoed to record. The degree of mucus secretion in lung tissue was analyzed by Image-pro plus 6.0 (Media Cybernetics, Inc., Rockville, MD, USA).

### ELISA and Cytokine Antibody Array

All ELISA operations, using Mouse IgE ELISA Kit, Mouse OVA sIgE ELISA Kit, Histamine ELISA Kit, and Mouse LTC4 ELISA Kit, were performed in strict accordance with the instructions. Measure the OD value of each well at 450 nm wavelength with a microplate reader.

Possible differential expressed proteins in mice serum samples were screened out by mouse cytokines antibody array 4000 kit, which was a combination of five non-overlapping arrays that could measure 200 kinds of mouse cytokines quantitatively. The operation was also performed in strict accordance with the instructions. The signal was scanned by a laser scanner with the Cy3 channel and excitation frequency of 532 nm. Cytokines antibody array analysis software (genepix, USA) was used to analyze the scanning results.

### Cell Culture and Cell Viability Test

RBL-2H3 cells were cultured in MEM with 100 U/ml penicillin, and 100 μg/ml streptomycin, contained 10% FBS. The necessary culture environment is 37°C, air containing 5% CO_2_ in the closed culture, which is provided by CO_2_ constant-temperature incubator. The number of RBL-2H3 cells in logarithmic growth phase were adjusted to 4 × 10^4^ cells/ml, after which loaded 100 μl/well into a 96-well plate, followed by a 12 h incubation. The blank control group with only culture medium was also set. When the incubation finished, stimulate cells 24 h by TMDCD of different concentrations, which was 0, 20, 40, 80 μg/ml, respectively. CCK-8 solution was added to each well after the stimulation, then measure the OD value of each well at 450 nm wavelength with a microplate reader when another 4 h of incubation finished.

Cell viability(%)=[A(Test group)−A(blank control group)]/[A(Test group 0)−A(blank control group)]×100%

### β-Hexosaminidase Release Assay

Loaded 5 × 10^4^ of RBL-2H3 cells in logarithmic growth phase into each well of 24-well plate, followed by a 12 h cell culture. 100 ng/ml of mouse DNP-IgE was added into each well of the plate, which was then incubated for 24 h in the environment of 37°C with 5% CO_2_, then discard the medium, rinsed 2 times by PIPES buffer (pH 7.2). Different concentrations of TMDCD (20, 40, and 80 μg/ml) and 100 nM of dexamethasone were then added into corresponded wells to stimulate the cells for 1 h. 100 ng/ml DNP-BSA per well was followed been added to react with the cells for 2 h before the supernatant was recycled (50 μl of the supernatant in each well was sucked out and transferred into a new 96 well plate, respectively). To lysis the cell, 250 μl Triton X-100 was added to each well for 5 min, and 50 μl of which was then was transferred to the a new 96 well plate mentioned above. The chambers on 96 well plate contained cell supernatant or lysis buffer were then mixed in 50 μl 0.1 M citrate buffer (pH 4.5) with 5 mM 4-nitrophenyl N-acetyl-β-Dglucosaminide, timed 1 h at room temperature, before 100 μl of stop reagent (0.1 M Na_2_CO_3_/NaHCO_3_, pH 10.0) being added. The OD value of each well at 405 nm was measured by a microplate reader.

Degranulation(%)=OD supernatant/(OD supernatant+OD lysed)×100%

### Toluidine Blue Staining

Cell culture, TMDCD and dexamethasone treatment, DNP-IgE/BSA stimulation were the same as above. Then they were washed with PBS twice. Dipped in 250 μl, 4% paraformaldehyde for 30 min at room temperature to fix them. Then the cells were stained with toluidine blue for 30 min, the color was separated with 95% ethanol for 5 min. Photoed and recorded using a microscope.

### Data Analysis and Statistics

Statistical analysis was performed using SPSS 20.0 statistical software (SPSS Inc., USA), the results were presented as Mean ± SEM (standard error of the mean). One-way ANOVA analysis was used to determine differences between every two groups among all, the *p* values of less than 0.05 were considered statistically significant. Using STRING (https://string-db.org/) and KEGG (https://www.kegg.jp/kegg/) to enrich and analyze the results of cytokines antibody array. All statistical analysis results were presented using GraphPad Prism 6.0 (GraphPad Software Inc., USA).

Images of GSM-CAA-4000 were scanned by an InnoScan 300 Microarray Scanner (Innopsys Inc., France). And using AAH-BLM data analysis software (Innopsys Inc., France) to analyze the obtained signal strength.

## Results

### Component Analysis of TMDCD

The chromatograms of positive and negative ions are shown in Supplementary Figure S2. According to the comparison with the database, 223 substances were identified (Total score >80). The substance categories in TMDCD include Phenylpropanoid, Flavonoids, Flavonoid glycosides, Cyanogenic glycosides, Triterpene saponins, Triterpenoids, Amino acids, Organic acids, etc. Specifically, the main substances include Ephedrine, Liquiritin, Glycyrrhizate, Isorhamnetin, Isorhamnetin-3-glucoside, Amygdalin, Pygenic acid B, Proline, Arginine, Phenylalanine, Emodin, Catechin, Gallic acid, Ferulic acid, etc. (Supplementary Table S2, S3). Most of these monomers can be found in the raw materials of TMDCD, as reported in literatures. And they are generally considered to have anti-inflammatory, anti-allergic and antiasthmatic effects.

### TMDCD Reduced AHR in OVA-Challenged Mice

**FIGURE 2:**
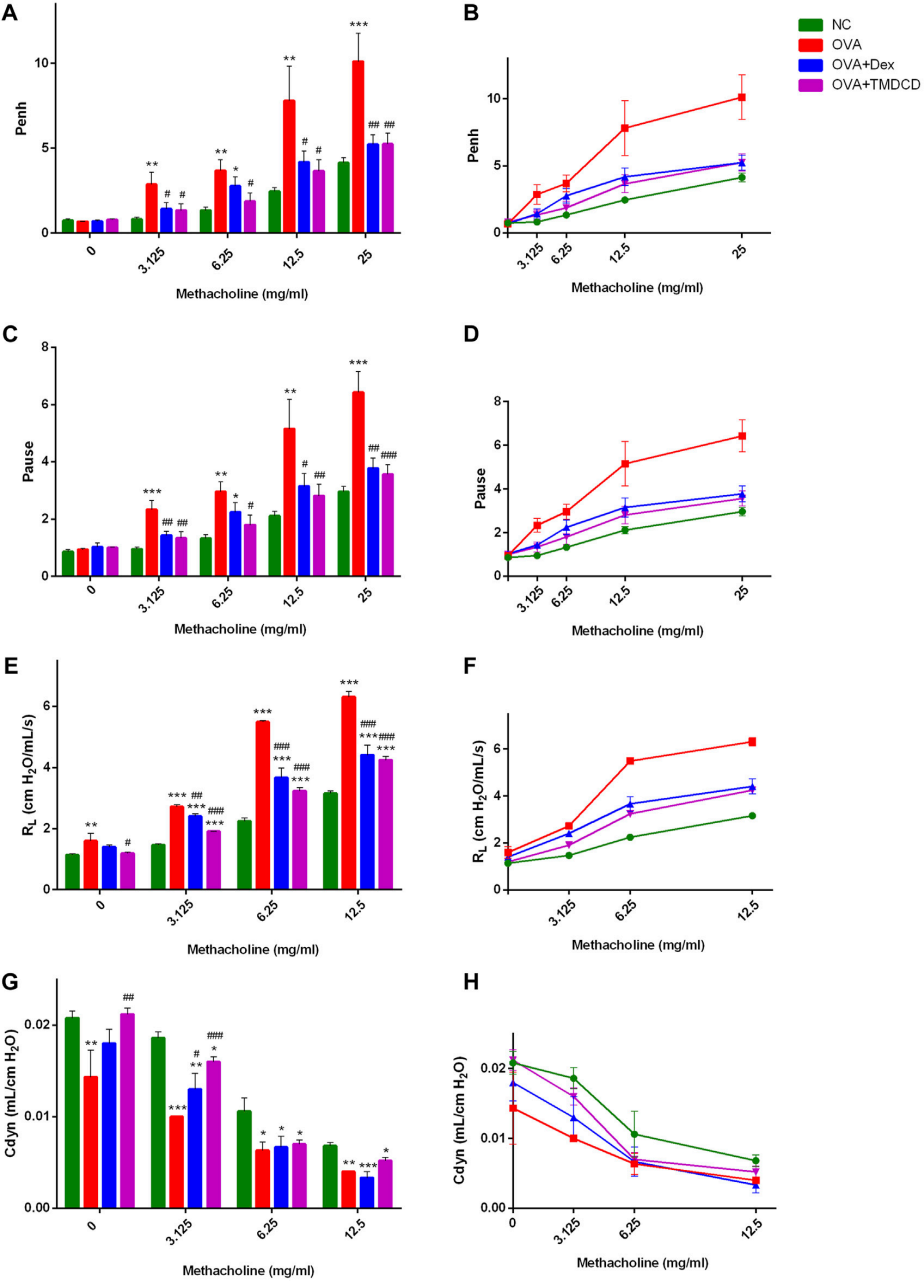
Detection of AHR. **(A, B)**, after stimulation with MeCh, the difference and overall trend of Penh value among the groups. **(C, D)**, after stimulation with MeCh, the difference and overall trend of Pause value among the groups. **(E, F)**, the difference and overall trend of R_L_ values among groups after MeCh stimulation. **(G, H)**, after stimulation with MeCh, the difference and overall trend of Cdyn values among groups. The results were expressed as Mean ± SEM, compared with NC group, *p < 0.05, *p < 0.01, *p < 0.001. Compared with OVA group, #*p* < 0.05, #*p* < 0.01, #*p* < 0.001. (WBPs: *n* = 5–6 mice/group, RCs: *n* = 3–5 mice/group. The results were from two independent experiments).

Excessive airway constriction is considered clinically an important feature of allergic asthma. It has been proved that OVA sensitization and challenge can induce AHR in mice (Reddy et al., 2012). The AHR results in our experiment indicated that, with the increase of MeCh concentration, the Penh value of mice in each group showed a gradually increasing trend, and the increase of mice Penh value in the OVA group was greater than that in other groups (Figure 2B). Compared with the NC group, there was no significant difference in the Penh value of mice in each group when they were not stimulated by MeCh (methacholine = 0 mg/ml), but with the increase of MeCh concentration, the Penh value of OVA group increased significantly (*p* < 0.05) (Figure 2A). Compared with the OVA group, the Penh values of mice in the OVA + TMDCD group were significantly decreased after being stimulated by different concentrations of MeCh (*p* < 0.05), and the change rules of the OVA + Dex group were basically the same as that of the OVA + TMDCD group (Figure 2A). The change rule of Pause value and R_L_ value under different concentrations of MeCh stimulation was basically consistent with that of Penh value (Figures 2C–F). The difference was that compared with the NC group, the baseline level of R_L_ in the OVA group was higher (*p* < 0.05) (Figure 2E). Generally speaking, when R_L_ value shows an upward trend, Cdyn value shows a corresponding downward trend. Our study also proved this rule. With the increase of MeCh concentration, the Cdyn value of mice in each group showed a gradually decreasing trend (Figure 2H). Compared with NC group, with the increase of MeCh concentration, the value of Cdyn in the OVA group all decreased significantly (*p* < 0.05) (Figure 2G). When MeCh concentration reached 6.25 mg/ml and 12.5 mg/ml, there was no significant difference in Cdyn between the OVA + Dex group and the OVA + TMDCD group (*p* > 0.05) (Figure 2G).

### TMDCD Reduced the Number of Eosinophils and Total IgE in BALF of OVA-Challenged Mice

**FIGURE 3:**
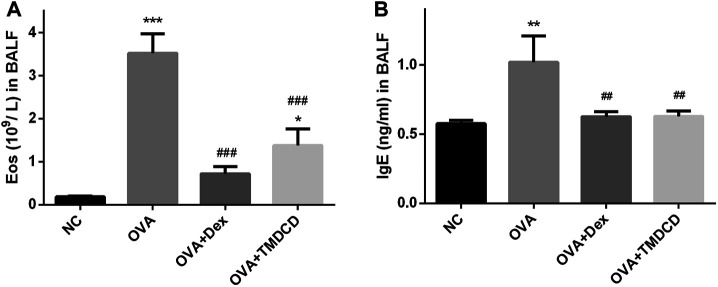
The number of eosinophils and IgE level in BALF. **(A)** Eosinophils count. **(B)** The level of IgE was detected by ELISA. The results were expressed as Mean ± SEM. Compared with NC group, **p < 0.01, ***p < 0.001, compared with OVA group, ##*p* < 0.01, ###*p* < 0.001 (*n* = 5 mice/group).

One of the clinical features of allergic asthma is the accumulation of eosinophils in lung tissue. We collected BALF and counted the eosinophils. Compared with the NC group, the number of eosinophils in BALF of the OVA group was significantly increased (*p* < 0.001) (Figure 3A). However, compared with the OVA group, the number of eosinophils in BALF decreased significantly after treatment with dexamethasone or TMDCD (*p* < 0.01), which is consistent with the Diff staining result of BALF inflammatory cells in our pre-experiment (Supplementary Figure S3). In addition, we also detected the total IgE level in BALF. The results showed that the level of total IgE in BALF of the OVA group was significantly higher than that of the NC group (*p* < 0.01), while the level of total IgE in BALF of the OVA + Dex group and the OVA + TMDCD group was significantly lower than that of the OVA group (*p* < 0.01).

### TMDCD Alleviated Lung Inflammation Accumulation in OVA-Challenged Mice

**FIGURE 4:**
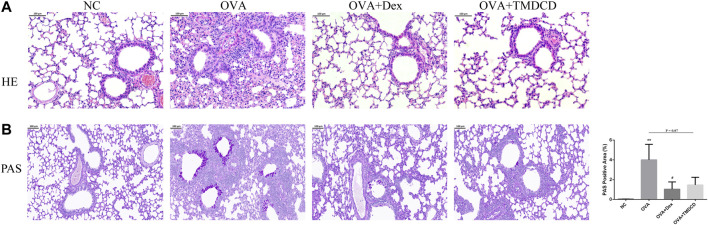
H&E and PAS staining of mice lung tissue. **(A)** Histopathology: infiltration of inflammatory cells around the bronchus (H&E staining, magnification×100). **(B)** Histopathology: secretion of mucus around bronchus (PAS staining, magnification×100). The results were expressed as Mean ± SEM. Compared with NC group, *p < 0.05, **p < 0.01, compared with OVA group, #*p* < 0.05 (*n* = 5 mice/group).

H&E staining and PAS staining were used to observe whether TMDCD could alleviate the lung inflammation of OVA-challenged mice. The results showed that no inflammatory cell infiltration and mucus secretion were observed in the lung tissue of the NC group, while the accumulation of inflammatory cells and airway mucus secretion in the peribronchial and perivascular areas of the OVA group were significantly increased (Figures 4A,B). Compared with the OVA group, after treatment with dexamethasone or TMDCD, inflammatory cell infiltration in OVA-challenged mice lung tissue was significantly reduced (Figure 4A), airway mucus secretion in the OVA + Dex group and the OVA + TMDCD group was decreased, and the degree of the OVA + Dex group was more significant (*p* < 0.05) (Figure 4B).

### TMDCD Regulated the Total IgE and OVA-specific IgE in Serum of OVA-Challenged Mice

**FIGURE 5:**
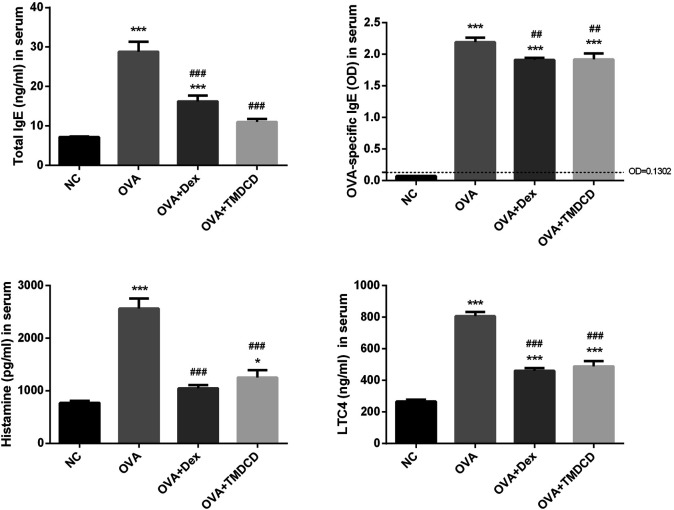
Total IgE, OVA specific IgE, histamine and LTC4 in serum were detected by ELISA. The results were expressed as Mean ± SEM. Compared with NC group, *p < 0.05, ***p < 0.001, compared with OVA group, ##*p* < 0.01, ###*p* < 0.001 (*n* = 5 mice/group).

Clinically, the serum total IgE level of patients with allergic asthma is usually higher than that of healthy people. The serum total IgE and other related mediators were detected by ELISA. The results showed that the total IgE, histamine and LTC4 levels in the OVA group were significantly increased compared to the NC group (*p* < 0.001) (Figure 5). Compared with the OVA group, the serum levels of total IgE, histamine and LTC4 were significantly lower after treatment with dexamethasone or TMDCD (Figure 5). According to the instructions, the OD value of the sample is compared with that of the control. If the OD value of the sample is more than 2.1 times of that of the negative control, the result is positive; If the OD of the sample is less than 2.1 times that of the negative sample, the result is negative. In this study, the 2.1 × OD_negative_ = 0.1302. The results of OVA-specific IgE positive test showed that OVA-specific IgE was positive in the serum of all groups except the NC group. However, compared with the OVA group, the OD value of OVA-specific IgE in the serum of mice treated with dexamethasone or TMDCD was significantly decreased (*p* < 0.01).

### Regulation Effects of TMDCD on Differential Expression Proteins in Serum

**FIGURE 6:**
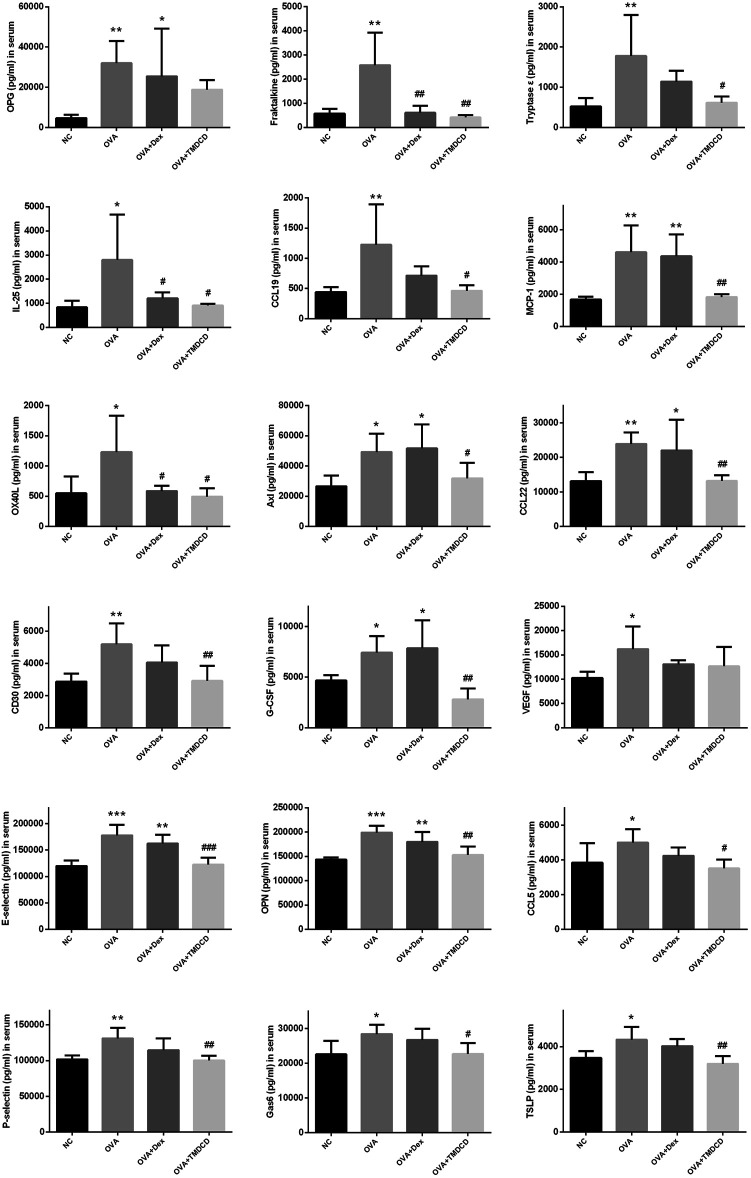
The regulation of TMDCD on serum differential expressed proteins. The results were expressed as Mean ± SEM. Compared with NC group, *p < 0.05, **p < 0.01, ***p < 0.001, compared with OVA group, #*p* < 0.5, ##*p* < 0.01, ###*p* < 0.001 (*n* = 4–5 mice/group).

Cytokines are a class of small molecular proteins with extensive biological activities synthesized and secreted by cells after being stimulated (Desai and Brightling, 2009). The inflammatory process of allergic asthma is coordinated by cytokines network. We used cytokines antibody array to explore the regulatory effect of TMDCD on cytokines in serum of OVA challenged mice. Based on the conditions of *p* < 0.05, fold change >1.2 or <0.83, and average signal value >150 (according to the manual recommendation), 18 up-regulated differential expression proteins in serum of the OVA group compared to the NC group were obtained (Table 2). The effects of dexamethasone and TMDCD on these differential expression proteins were observed. Under the conditions of *p* < 0.05 and fold change <0.83, compared with the OVA group, Fractalkine, IL-25 and OX40L in serum of the OVA + Dex group were significantly down-regulated, and other differential expression proteins except for OPG and VEGF in serum of the OVA + TMDCD group were significantly down-regulated (Table 3). The analysis and comparison of the specific expression levels of 18 differential expression proteins are shown in Figure 6.

**TABLE 2:** Relative expression changes of differential expressed proteins in serum of OVA-challenged mice (*n* = 4–5).

Uniprot ID	Protein ID	OVA group/NC group fold change	P
O08712	OPG	6.79	0.009
O35188	Fractalkine	4.52	0.002
Q9ER10	Tryptase ε	3.40	0.007
Q8VHH8	IL-25	3.34	0.016
O70460	CCL19	2.77	0.009
P10148	MCP-1	2.77	0.002
P43488	OX40L	2.24	0.015
Q00993	Axl	1.86	0.012
O88430	CCL22	1.82	0.006
Q60846	CD30	1.80	0.005
P09920	G-CSF	1.59	0.030
Q00731	VEGF	1.58	0.018
Q00690	E-selectin	1.48	0.000
P10923	OPN	1.39	0.000
P30882	CCL5	1.30	0.042
Q01102	P-selectin	1.29	0.003
Q61592	Gas6	1.26	0.018
Q9JIE6	TSLP	1.25	0.011

**TABLE 3:** Effect of TMDCD on relative expression of differential expressed proteins in serum of OVA-challenged mice (n = 4-5 mice/group).

Protein ID	OVA + Dex group/OVA group fold change	P	OVA + TMDCD group/OVA group fold change	P
OPG	0.80	0.472	0.59	0.161
Fractalkine	0.24	0.002	0.16	0.001
Tryptase ε	0.64	0.131	0.35	0.012
IL-25	0.43	0.043	0.33	0.020
CCL19	0.58	0.068	0.38	0.011
MCP-1	0.95	0.746	0.40	0.003
OX40L	0.47	0.020	0.40	0.010
Axl	1.05	0.768	0.65	0.046
CCL22	0.92	0.567	0.55	0.006
CD30	0.78	0.124	0.56	0.006
G-CSF	1.06	0.709	0.38	0.001
VEGF	0.81	0.176	0.78	0.130
E-selectin	0.91	0.164	0.69	0.000
OPN	0.90	0.082	0.77	0.001
CCL5	0.85	0.165	0.70	0.012
P-selectin	0.87	0.060	0.76	0.002
Gas6	0.94	0.456	0.80	0.019
TSLP	0.93	0.315	0.74	0.002

### Enrichment Analysis of Serum Differential Expression Proteins in OVA-Challenged Mice

**FIGURE 7:**
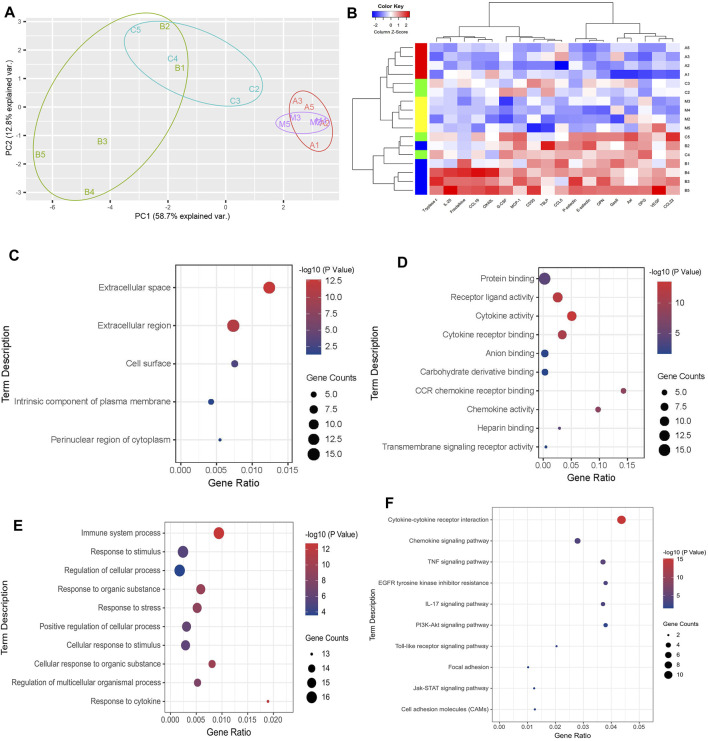
**(A)** Principal component analysis of differential expressed proteins. **(B)** Clustering analysis of differential expression proteins, A: NC group, B: OVA group, C: OVA + DEX group, D: OVA + TMDCD group. The color key indicates the protein expression level: red represents high-level expression, blue represents low-level expression. **(C)** GO pathway enrichment analysis of CC. **(D)** GO pathway enrichment analysis of MF (top 10). **(E)** GO pathway enrichment analysis of BP (top 10). **(F)** KEGG pathway enrichment analysis.

PCA analysis of the above 18 differential expression proteins showed that the aggregation phenomenon was obvious in each group. Among them, the NC group, the OVA + TMDCD group were significantly different from the OVA group (Figure 7A). Furthermore, cluster analysis of 18 differential expression proteins was carried out to determine whether the differential expression proteins can be used to classify each group. The results showed that the clustering of the NC group, the OVA group and the OVA + TMDCD group was obvious, especially the NC group and the OVA group were obviously distinguished, which was basically consistent with PCA analysis results (Figure 7B).

To further understand the biological process of differential expression proteins, KEGG pathway enrichment analysis and GO enrichment analysis were carried out. GO enrichment analysis classified and annotated the differential expression proteins according to the gene functions of cellular component (CC), molecular function (MF) and biological process (BP). Among them, CC results showed that most of the differential expression proteins existed in extracellular tissue fluid and blood (Figure 7C, Supplementary Table S9). MF results showed that the main molecular functions of differential expression proteins included protein binding, receptor-ligand activity, cytokines activity and cytokines receptor binding (Figure 7D, Supplementary Table S10). BP results showed that the main biological processes of differential expression proteins included immune system process, response to stimulus and regulation of cellular process (Figure 7E, Supplementary Table S11). KEGG pathway enrichment analysis revealed chemokine signaling pathway, TNF signaling pathway, and IL-17 signaling pathway, PI3K-Akt signaling pathway, etc. (Figure 7F, Supplementary Table S12).

### TMDCD Significantly Suppresses Mast Cell Degranulation

**FIGURE 8:**
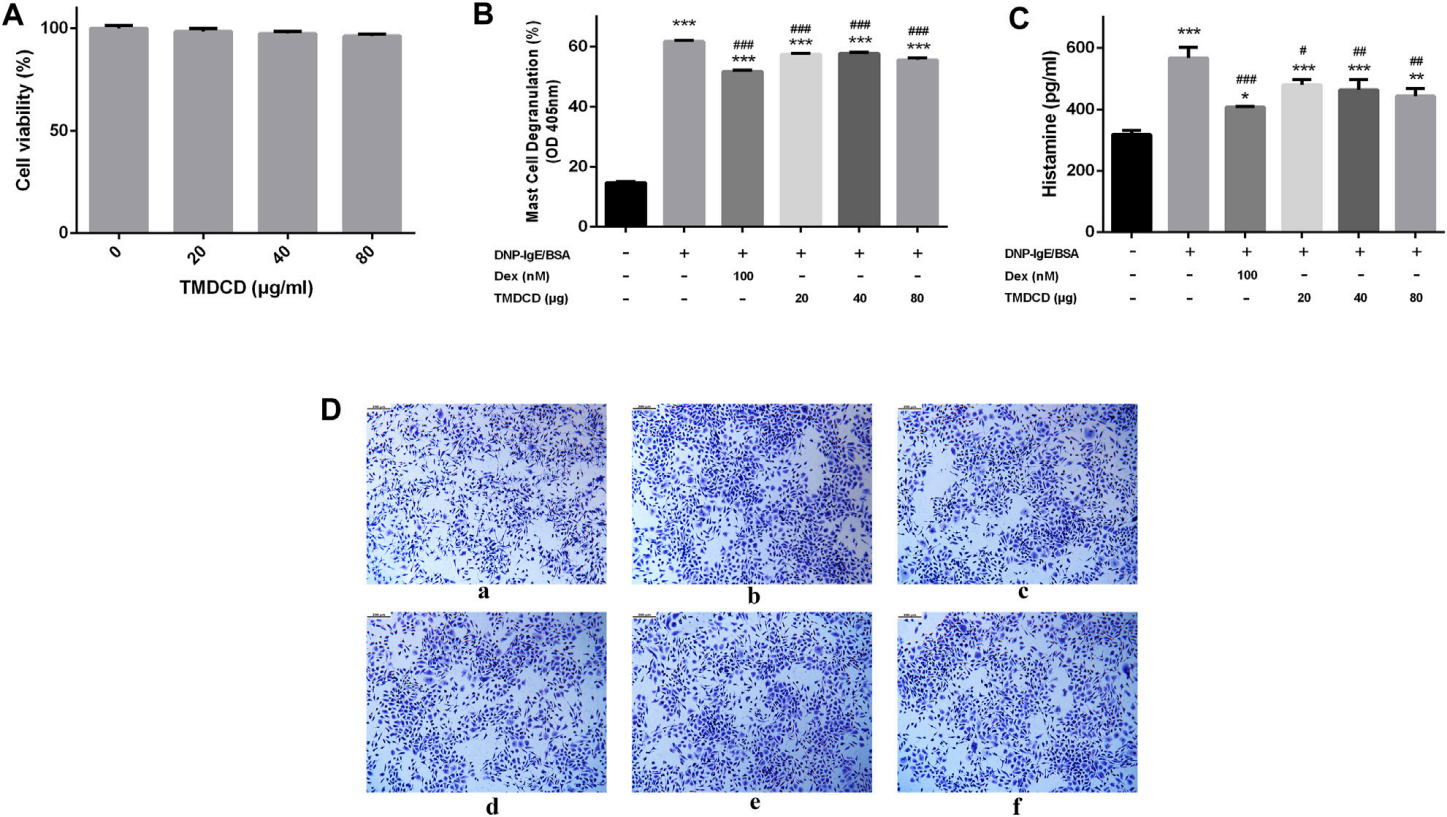
Effect of TMDCD on degranulation of mast cells. **(A)** Effect of TMDCD on the viability of RBL-2H3 cells. **(B)** Effect of TMDCD on the release of β-hexosaminidase. The results were expressed as Mean ± SEM. **(C)** Effect of TMDCD on histamine release. **(D)** Effect of TMDCD on morphology of RBL-2H3 cells sensitized by DNP-IgE/BSA. a. Blank group; b. Model group; c. Dexamethasone group; d. TMDCD 20 μg/ml group; e. TMDCD 40 μg/ml group; f. TMDCD 80 μg/ml group (toluidine blue staining, original magnification ×100). Compared with the control group, ***p < 0.001, compared with the model group, #*p* < 0.05, ##*p* < 0.01 and ###*p* < 0.001.

MC exists in almost all vascularized tissues and is a potential source of many bioactive secretory products, including cytokines and growth factors (Mukai et al., 2018). The differential expression proteins found in animal experiments are directly or indirectly related to the activation and recruitment of MC, for example, MCP-1, CCL5 and OPN can be released from MC (Zhang et al., 2011; Mukai et al., 2018; Paivandy and Pejler, 2021). Therefore, we speculated that TMDCD may inhibit MC degranulation. We evaluated the effect of TMDCD on RBL-2H3 cell viability by CCK-8 to ensure that the decrease of MC degranulation level was not due to the cell death. The results showed that TMDCD at a concentration less than 80 μg/ml did not induce cytotoxicity to RBL-2H3 cells in 24 h (Figure 8A). We then investigated the inhibitory effect of TMDCD on degranulation of mast cells sensitized by DNP-IgE/BSA *via* measuring the release level of β-hexosaminase. The results showed that the degranulation rate of mast cells increased significantly after sensitization with DNP-IgE/BSA (degranulation rate = 61.68%; *p* < 0.001). However, the degranulation rate of MC treated with dexamethasone (51.70%, *p* < 0.001) and TMDCD (20 μg/ml degranulation rate = 57.46%, 40 μg/ml degranulation rate = 57.69%, 80 μg/ml degranulation rate = 55.57%; *p* < 0.001) decreased significantly (Figure 8B). The release level of histamine was consistent with that of degranulation (Figure 8C). Toluidine blue staining showed that the morphology of RBL-2H3 cells in the blank group was elongated fusiform and dark purple. After sensitized by DNP-IgE/BSA, the cells in the model group showed the irregular shape and increased volume, and the granular substances in the cells were released, the situation has alleviated significantly when the cells were intervened by dexamethasone or TMDCD (Figure 8D).

## Discussion

With the increasing incidence rate of asthma in both children and adults, it has become a public health problem that reducing patients’ life quality seriously. In China, there are about 45.7 million people over 20 years old with asthma, of which nearly 70% have not been diagnosed and 95% have not received standard treatment. China is facing a severe burden of asthma and related diseases (Huang et al., 2019). At present, the regular use of corticosteroids is still a common treatment for asthma, despite its obvious side effects. Some other treatment methods, such as targeted therapy (E.g, monoclonal antibodies against IgE, anti-IL-5 and anti-IL-5 receptor therapies), and allergen-specific immunotherapy have been proposed and used in the clinic, but the disadvantages are their limitation and high cost of treatment (Cevhertas et al., 2020). In recent years, researchers have found that some traditional Chinese medicine or their extracts have a better effect in the treatment of asthma, and may bring higher cost-effectiveness. However, the mechanism of Chinese herbal compounds is complex, always with multiple targets and pathways (Song et al., 2018). TMDCD in this study was a Chinese herbal compound composed of 12 medicinal materials under the guidance of the theory diagnosis and treatment model of constitution-disease differentiation, which shows a good effect in the clinic but a lack in mechanism research. We used OVA-induced allergic asthma mice, a model that has been widely used in the study of allergic asthma as the model, to study the mechanism of TMDCD on allergic asthma. This study confirmed that TMDCD can alleviate lung inflammation and airway remodeling, reduce airway AHR and reduce IgE mediated allergic reaction *in vivo* experiments of allergic asthma mice, and found that TMDCD can regulate the expression of a variety of differentially expressed proteins in the serum of allergic asthma mice. After that, we confirmed that TMDCD could inhibit IgE mediated MC degranulation *in vitro*.

Asthma is characterized by typical daily symptoms such as dyspnea, cough and wheezing, accompanied by acute exacerbation, variable airflow obstruction and AHR. These typical symptoms and airway dysfunction occur in the context of airway inflammation and remodeling (Desai and Brightling, 2009). Meanwhile, repeated allergic pulmonary inflammation can cause changes in cell and extracellular matrix, apoptosis of epithelial cells, the proliferation of airway smooth muscle cells and activation of fibroblasts, which lead to airway remodeling (Hough et al., 2020). In this study, we used WBPs and RCs to evaluate AHR of experimental animals. The results of two evaluation systems showed that AHR of allergic asthma mice was significantly alleviated after TMDCD treatment (Figure 2). In addition, we observed the H&E staining and PAS staining of lung tissue in mice. We also found that the hypertrophy and proliferation of goblet cells, airway wall thickening, airway stenosis, airway mucus secretion of allergic asthma mice were significantly alleviated after TMDCD treatment, and the infiltration of inflammatory cells in bronchial and perivascular areas was also alleviated significantly (Figure 4). At the same time, TMDCD treatment can significantly reduce the number of eosinophils in BALF of allergic asthma mice (Figure 3A). These results suggest that the antiasthmatic effect of TMDCD may be achieved by inhibiting airway remodeling through anti-inflammatory effect.

Exploring disease biomarkers can help to monitor the progress of disease and disease treatment. Cytokines have a wide range of biological significance. The interaction between cytokines and cells can transmit signals between cells in asthma, and participate in a variety of biological processes, including inflammatory cell recruitment, airway remodeling, Th2 cell polarization, IgE synthesis, mast cell degranulation, etc. These cytokines driven effects are used to connect the complex network of cell-cell interactions in asthma (Lambrecht et al., 2019). Therefore, this study used cytokines antibody array to explore the changes of cytokines in serum of allergic asthma mice, and finally screened out 18 differential expression proteins in serum of allergic asthma mice, which were OPG, Fractalkine, Tryptase ε, IL-25, CCL19, MCP-1, OX40L, Axl, CCL22, CD30, G-CSF, VEGF, E-selectin, OPN, CCL5, P-selectin, Gas6, TSLP. Among them, MCP-1, G-CSF (Kato et al., 2010), CD30 (Lombardi et al., 2010), CCL5 (Hamsten et al., 2016), E-selectin (Bi et al., 2018), P-selectin (Johansson et al., 2020), OPN (Xu et al., 2019) have been repeatedly reported as biomarkers of allergic asthma, which were highly expressed in the serum of patients.

Through this study, we noticed that TMDCD can down-regulate 16 differential expressed proteins such as Fractalkine, Tryptase ε, IL-25, CCL19, MCP-1, OX40L, Axl, CCL22, CD30, G-CSF, E-selectin, OPN, CCL5, P-selectin, Gas6, and TSLP, which may be the key target of TMDCD in the treatment of allergic asthma. On this basis, we annotated the function of these differentially expressed proteins by GO enrichment analysis, and found that most of the 16 differentially expressed proteins down-regulated by TMDCD were involved in regulating the differentiation and chemotaxis of a variety of immune cells. Therefore, we speculate that TMDCD may play anti-inflammatory and immunomodulatory roles by regulating these biological processes (table S11). For example, CD30, a costimulatory molecule of TNF receptor superfamily, exists on the surface of different immune cells or in serum in soluble form (Horie and Watanabe, 1998). There is direct evidence that CD30 signaling is crucial for the production of Th2 cytokines and IgE in the mouse model of allergic asthma (Polte et al., 2006). In this study, TMDCD can significantly down-regulate CD30 in serum of allergic asthma mice, which may be the reason for the decrease of serum IgE level after its intervention. CCL22 can bind to CCR4 receptor on Th2 cell surface and induce selective migration of Th2 cells to be recruited into airway (Garcia et al., 2005). OPN can enhance the degranulation and migration of MC mediated by IgE, and enhance the response of MC to antigens (Nagasaka et al., 2008). TSLP is mainly released by epithelial cells under the action of pro-inflammatory environment and trypsin. It can activate dendritic cells and induce Th2 type immune response, bind to TSLP receptor (TSLPR) on the surface of CD4^+^T cells to induce Th2 type immune response directly, inhibit the activity of pulmonary Treg cells and aggravate inflammation, and can also activate eosinophils and mast cells to increase the production of Th2 cytokines (West et al., 2012). Both E-selectin and P-selectin are endothelial cell adhesion molecules, which can be released from the cell surface by proteolysis into extracellular fluid to form serum soluble E—or soluble P-selectin (Bi et al., 2018). Some studies have found that MC progenitor cells can roll on skin vessels under the action of E—or P-selectin, and show strong adhesion to skin endothelial cells under static and flow conditions *in vitro*, which is one of the reasons for the increase of mast cells in chronic inflammation (Dudeck et al., 2010). Conversely, activated mast cells can release a variety of pro-inflammatory cytokines and induce the expression of E-selectin and P-selectin (Zhang et al., 2011). Combined with our results, whether TMDCD can reduce the recruitment of MC in the lung by reducing E-and P-selectin, so as to play a role in relieving the symptoms of allergic asthma still needs to be further explored. Tryptase ε mainly exists in mouse mast cells, which can promote the proliferation and contraction of airway smooth muscle cells, stimulate lung fibroblasts to produce collagen, and degrade muscle relaxant neuropeptides (Wong and Stevens, 2005; Berger et al., 2001). The main source of CCL19 is airway smooth muscle cells and mast cells, especially in asthmatic patients with mast cells, showing high expression of CCL19, which can mediate the migration and repair of airway smooth muscle in asthma (Kaur et al., 2006). MCP-1 regulates the migration and infiltration of inflammatory monocytes, memory T lymphocytes and MC(Deshmane et al., 2009). CCL5 can promote the recruitment of MC to airway epithelium and participate in local inflammatory response (Bradding and Arthur, 2016). In addition, some studies have found that MC activated by IL-33 can release CCL5 and MCP-1 through MAPK and NF-κB signaling pathways (Bawazeer and Theoharides, 2019). In the immune system, MC can act as a link between innate immunity and adaptive immunity. In this part of the study, many of the differentially expressed proteins we screened were directly or indirectly related to MC. For example, tryptase ε, Histamine and LTC4 are mainly generated from MC, while CCL19, CCL22, MCP-1, OPN and CCL5 can also be partially generated by MC. This aroused our curiosity: can TMDCD inhibit MC degranulation? In subsequent *in vitro* experiments, we found that TMDCD did have a considerable inhibitory effect on IgE mediated MC degranulation, but the specific mechanism needs to be further studied. Our researchers are also continuing this part of the research.

There may be direct or indirect links between these differentially expressed proteins, we used KEGG enrichment analysis to predict the signal pathways that differentially expressed proteins may participate in. In KEGG pathway enrichment analysis, the differential expression proteins were enriched in chemokine signaling pathway, TNF signaling pathway, and IL-17 signaling pathway, PI3K-Akt signaling pathway (Figure 7C, table S12), which has been reported closely related to asthma. TNF signaling pathway has a variety of biological effects. It can regulate a variety of intracellular signaling pathways, affect cell apoptosis, cell survival, inflammation and immunity, and participate in the occurrence of asthma (Krusche et al., 2019). IL-17 signaling pathway plays an important role in both acute and chronic inflammatory responses, and NF-κB and MAPK are its downstream signals (Song and Qian, 2013). PI3K-Akt signaling pathway is a key role in innate immunity and adaptive immunity (Fruman et al., 2017). It has been found that PI3K-Akt signaling pathway is related to airway remodeling in asthma (Shao et al., 2019; Xing et al., 2021). In the process of asthma, the activation of PI3K-Akt signaling pathway can lead to the proliferation, migration and phenotypic transformation of airway smooth muscle cells stimulated by platelet derived growth factor BB(Pang and Qiao, 2020). These signal pathways are only based on the speculation of KEGG enrichment analysis. In which signal pathways TMDCD plays a role needs to be further studied. It can also be inferred that TMDCD may play a role in the treatment of allergic asthma through the synergistic effect of multiple chemical components.

The characteristic of this study is that the time point of administration of Chinese herbal compounds was specially considered. Dexamethasone, a corticosteroid, was selected as the positive control. Clinically, long-term oral administration of dexamethasone can cause many adverse reactions. Therefore, we chose to give dexamethasone 1 h before OVA challenge (as usual in most studies). TMDCD, a traditional Chinese medicine compound, was made up following the principle of diagnosis and treatment model of constitution-disease differentiation. In its clinical application, the strategy of preventive intervention is usually carried out, obeying the principle of “preventive treatment of disease,” therefore, in the design of this experiment, we chose to give TMDCD after the second OVA sensitization.

Our research also has some limitations, which need further research. For example, TMDCD used in this study is still a traditional Chinese medicine decoction. Although quality control has been carried out as far as possible in the preparation process, the content of effective chemical components may change due to small changes in the extraction method. Researchers may develop a more stable dosage form for subsequent research and application. Moreover, although we have proved there is a good therapeutic effect on allergic asthma, TMDCD, as a combination of 12 herbal medicines, contains many chemical components and has complex targets, still remains to study the chemical composition in more detail. More than that, we should further explore the mechanism of TMDCD on allergic asthma at the cellular and genetic levels according to the potential pathways revealed by differential expression proteins, combined with more *in vivo* and *in vitro* experiments. Our current work is to study the regulatory mechanism of TMDCD on allergic asthma by using the method of network pharmacology. Although TMDCD is composed of a variety of medicinal materials, its formula idea covers the concepts of diagnosis and treatment model of constitution-disease differentiation. So in the future, the components can be studied in the form of independent or small drug pairs, and its action mechanism can be further clarified.

## Conclusion

TMDCD can reduce the content of total IgE and OVA-specific IgE in serum of mice with allergic asthma. At the same time, TMDCD can reduce the levels of MC related proteins such as histamine, LTC4 and Tryptase ε in the serum of allergic asthma mice. *In vitro* experiments showed that TMDCD could inhibit IgE mediated degranulation and histamine release of RBL-2H3 cells, indicating that TMDCD had anti allergic effect. *In vivo* experiments show that TMDCD can reduce lung inflammation and alleviate airway remodeling in allergic asthmatic mice, which proves that TMDCD has anti-inflammatory and antiasthmatic effects.

## Data Availability

The datasets presented in this study can be found in online repositories. The names of the repository/repositories and accession number(s) can be found in the article/Supplementary Material.
