# Supplementary material for: Tuo-Min-Ding-Chuan Decoction Alleviate Ovalbumin-Induced Allergic Asthma by Inhibiting Mast Cell Degranulation and Down-Regulating the Differential Expression Proteins
Source: Front Pharmacol. 2021 Sep 22;12:725953. doi: 10.3389/fphar.2021.725953 (PMC8493414; doi:10.3389/fphar.2021.725953)
Supplement: Supplementary file 1 [file DataSheet1.doc]

Article Material for

# Tuo-Min-Ding-Chuan Decoction alleviate ovalbumin-induced allergic asthma by inhibiting mast cell degranulation and decreasing differential proteins expression

Jingbo Qin^1^, Mingsheng Lv^2^, Zeqiang Jiang^1^, Xianghe Meng^1^, Yi Wang^1^, Jiarui Cui^1^, Ji Wang ^1*^, Qi Wang^1*^

^1^School of Traditional Chinese Medicine, Beijing University of Chinese Medicine (BUCM) , Beijing, China

^2^Respiratory Department, BUCM Third Affiliated Hospital, Beijing, China

List of supplementary materials

| No. | Content | Figure/Table |
| --- | --- | --- |
| 1 | Chromatographic Conditions | Table S1 |
| 2 | TMDCD chemical composition - LC-MS positive ion mode | Table S2 |
| 3 | TMDCD chemical composition - LC-MS negative ion mode | Table S3 |
| 4 | AHR test | Table S4 |
| 5 | Eosinophils count and IgE level in BALF | Table S5 |
| 6 | PAS positive area in lung tissue | Table S6 |
| 7 | Total IgE, OVA-specific IgE, Histamine, LTC4 of serum | Table S7 |
| 8 | Differentially expressed proteins of serum | Table S8 |
| 9 | GO (CC) enrichment analysis of differentially expressed protein | Table S9 |
| 10 | GO (MF) enrichment analysis of differentially expressed protein | Table S10 |
| 11 | GO (BP) enrichment analysis of differentially expressed protein | Table S11 |
| 12 | KEGG signaling pathway enrichment analysis of differentially expressed protein | Table S12 |
| 13 | Viability of RBL-2H3 cells | Table S13 |
| 14 | The release of β-hexosaminidase in RBL-2H3 cells | Table S14 |
| 15 | The release of histamine in RBL-2H3 cells | Table S15 |
| 16 | Traditional Chinese medicine in TMDCD | Fig. S1 |
| 17 | LC-MS positive and negative ion chromatogra | Fig. S2 |
| 18 | Diff staining of inflammatory cells in BALF | Fig. S3 |

1. Chromatographic Conditions

| Table S1 Chromatographic Conditions | |
| --- | --- |
| Time (min) | Mobile phase ratio |
| 0 | A: 5% B: 95% |
| 2 | A: 5% B: 95% |
| 4 | A: 20% B: 80% |
| 12 | A: 15% B:75% |
| 14 | A: 46% B: 54% |
| 26 | A: 100% B: 0% |
| 28 | A: 100% B: 0% |
| 29 | A: 5% B: 95% |
| 30 | A: 5% B: 95% |

1. TMDCD chemical composition - LC-MS positive ion mode

| Table S2 TMDCD chemical composition - LC-MS positive ion mode | | | | | | |
| --- | --- | --- | --- | --- | --- | --- |
| Component | Molecular formula | Ontology | RT  (min) | Height | Area | Total  score |
| Ephedrine | C10H15NO | Phenylpropanes | 5.5 | 3276278.5 | 23954504.0 | 89.0 |
| Proline | C5H9NO2 | Proline and derivatives | 1.4 | 1204387.5 | 9162814.0 | 87.1 |
| Arginine | C6H14N4O2 | L-alpha-amino acids | 1.3 | 962066.4 | 8091732.5 | 93.6 |
| DL-Pipecolinic acid | C6H11NO2 | Alpha amino acids | 2.0 | 549331.5 | 6664317.5 | 83.7 |
| Formononetin-7-O-glucoside | C22H22O9 | Isoflavonoid O-glycosides | 11.7 | 1020531.8 | 5554745.5 | 81.4 |
| Vitexin-2''-O-rhamnoside | C27H30O14 | Flavonoid 8-C-glycosides | 6.7 | 1161131.8 | 4172430.3 | 81.6 |
| isoliquiritigenin | C15H12O4 | 2'-Hydroxychalcones | 7.4 | 860181.8 | 3638533.8 | 86.4 |
| Adenosine | C10H13N5O4 | Purine nucleosides | 3.4 | 900627.1 | 3586175.8 | 84.4 |
| Phenylalanine | C9H11NO2 | Phenylalanine and derivatives | 3.7 | 856205.8 | 3492448.0 | 83.6 |
| Ephedrine | C10H15NO | Phenylpropanes | 5.5 | 3276278.5 | 23954504.0 | 89.0 |
| (2S)-4-hydroxy-2-(2-hydroxypropan-2-yl)-7-methyl-2,3-dihydrofuro[3,2-g]chromen-5-one | C15H16O5 | Furanochromones | 16.4 | 1015584.9 | 3397948.8 | 81.5 |
| Glycine-Betaine | C5H11NO2 | Alpha amino acids | 1.3 | 435691.4 | 3037948.3 | 93.0 |
| Choline | C5H14NO | Cholines | 1.3 | 378990.8 | 2920609.0 | 82.7 |
| Adenine | C5H5N5 | 6-aminopurines | 2.2 | 858172.9 | 2666715.8 | 87.0 |
| Liquiritin | C21H22O9 | Flavonoid O-glycosides | 11.1 | 434720.2 | 2368995.8 | 83.6 |
| 2-[4-[3-[3,4-dihydroxy-4-(hydroxymethyl)oxolan-2-yl]oxy-4,5-dihydroxy-6-(hydroxymethyl)oxan-2-yl]oxyphenyl]-7-hydroxy-2,3-dihydrochromen-4-one | C26H30O13 | Flavonoid O-glycosides | 7.2 | 582314.9 | 2357521.3 | 80.4 |
| Valine | C5H11NO2 | Valine and derivatives | 1.6 | 253127.1 | 2276577.5 | 80.0 |
| L-5-Oxoproline | C5H7NO3 | Alpha amino acids and derivatives | 1.8 | 448394.3 | 1892512.1 | 86.8 |
| Leucine | C6H13NO2 | Leucine and derivatives | 2.6 | 509895.1 | 1807516.3 | 89.8 |
| Phenylpropanolamine | C9H13NO | Phenylpropanes | 5.2 | 478475.1 | 1792959.3 | 86.7 |
| Isofraxidin | C11H10O5 | 7-hydroxycoumarins | 7.9 | 401264.3 | 1594639.0 | 81.2 |
| L-Carnitine | C7H15NO3 | Carnitines | 1.3 | 155888.1 | 1501818.1 | 92.8 |
| Tyrosine | C9H11NO3 | Tyrosine and derivatives | 2.4 | 452008.9 | 1466346.8 | 94.0 |
| Licoricesaponin G2 | C42H62O17 | Triterpene saponins | 15.5 | 414965.4 | 1428948.8 | 88.4 |
| (2S)-{[6-O-(beta-D-Glucopyranosyl)-beta-D-glucopyranosyl]oxy}(phenyl)acetonitrile | C20H27NO11 | Cyanogenic glycosides | 5.9 | 254059.1 | 1327147.1 | 84.0 |
| Guanosine | C10H13N5O5 | Purine nucleosides | 2.9 | 429090.6 | 1280174.3 | 85.7 |
| Trigonelline | C7H7NO2 | Alkaloids and derivatives | 1.3 | 183376.0 | 1243900.8 | 92.4 |
| Hordenine | C10H15NO | Phenethylamines | 3.4 | 310005.9 | 1176399.4 | 82.1 |
| 2-O-rhamnosyl-swertisin | C28H32O14 | Flavonoid C-glycosides | 11.3 | 214665.2 | 1173961.3 | 82.4 |
| icariside B5 | C19H32O8 | Fatty acyl glycosides of mono- and disaccharides | 6.2 | 351297.7 | 1111621.4 | 84.2 |
| Tryptophan | C11H12N2O2 | Indolyl carboxylic acids and derivatives | 5.1 | 342875.2 | 1057295.6 | 91.4 |
| DEOXYCARNITINE | C7H15NO2 | Straight chain fatty acids | 1.5 | 120582.9 | 1015905.3 | 84.5 |
| Glycyrrhizate | C42H62O16 | Triterpene saponins | 15.8 | 278509.3 | 992985.8 | 86.9 |
| N,N-Dimethylarginine | C8H18N4O2 | Alpha amino acids | 1.6 | 120737.3 | 915415.4 | 81.4 |
| 4-Guanidinobutyric acid | C5H11N3O2 | Gamma amino acids and derivatives | 2.2 | 314401.3 | 859621.1 | 89.4 |
| Xanthotoxin | C12H8O4 | 8-methoxypsoralens | 15.5 | 220530.9 | 807180.9 | 88.7 |
| Scopoletin | C10H8O4 | 7-hydroxycoumarins | 7.6 | 183634.1 | 752204.4 | 89.9 |
| 7-hydroxy-3,5-dimethylisochromen-1-one | C11H10O3 | Isocoumarins and derivatives | 10.4 | 122682.1 | 689616.6 | 83.4 |
| Isoflavone base + 1O, 1MeO, O-Hex+C7H12NO | C29H33NO10 | NA | 12.4 | 105627.4 | 643578.1 | 84.4 |
| Phenethylamine | C8H11N | Phenethylamines | 5.3 | 172941.4 | 611005.1 | 86.9 |
| Chlorogenic Acid | C16H18O9 | Quinic acids and derivatives | 5.4 | 205354.1 | 610414.8 | 82.7 |
| Biochanin-7-O-glucoside | C22H22O10 | Isoflavonoid O-glycosides | 14.8 | 159459.0 | 602056.0 | 80.0 |
| Cytidine | C9H13N3O5 | Pyrimidine nucleosides | 2.2 | 209870.6 | 584058.8 | 80.2 |
| Pyridoxamine | C8H12N2O2 | Pyridoxamine 5'-phosphates | 3.9 | 124889.1 | 548348.3 | 86.5 |
| Vitexin | C21H20O10 | Flavonoid 8-C-glycosides | 7.0 | 144948.3 | 536076.6 | 82.7 |
| 2-HYDROXYPYRIDINE | C5H5NO | Pyridinones | 2.1 | 111122.3 | 516986.4 | 82.3 |
| Ononin | C22H22O9 | Isoflavonoid O-glycosides | 14.7 | 150075.9 | 515069.6 | 84.7 |
| Phthalic anhydride | C8H4O3 | Phthalic anhydrides | 22.2 | 101301.9 | 512441.3 | 81.6 |
| Hypoxanthine | C5H4N4O | Hypoxanthines | 2.3 | 174952.8 | 504854.1 | 86.8 |
| Targinine | C7H16N4O2 | Arginine and derivatives | 1.4 | 70043.6 | 470770.8 | 80.3 |
| Guanosine-3',5'-cyclic monophosphate | C10H12N5O7P | 3',5'-cyclic purine nucleotides | 2.5 | 156567.5 | 470046.8 | 90.4 |
| 4-(2 AMINOETHYL)-PHENOL | C8H11NO | Phenethylamines | 2.9 | 142309.5 | 469542.8 | 93.0 |
| Nicotinamide | C6H6N2O | Nicotinamides | 2.8 | 140949.2 | 465268.1 | 85.7 |
| 3',5'-Cyclic AMP | C10H12N5O6P | 3',5'-cyclic purine nucleotides | 2.7 | 143761.6 | 439413.0 | 81.5 |
| D-(+)-Pantothenic acid | C9H17NO5 | Secondary alcohols | 4.6 | 116510.4 | 387426.9 | 82.4 |
| N'-(2,4-Dimethylphenyl)-N-methylformamidine | C10H14N2 | m-Xylenes | 4.1 | 85795.5 | 386533.0 | 80.4 |
| Guanine | C5H5N5O | Purines and purine derivatives | 2.2 | 127363.1 | 360248.3 | 86.4 |
| beta-D-Glucopyranoside, phenylmethyl 6-O-[(2R,3R,4R)-tetrahydro-3,4-dihydroxy-4-(hydroxymethyl)-2-furanyl]- | C18H26O10 | O-glycosyl compounds | 5.9 | 106974.2 | 356947.8 | 80.8 |
| Bergapten | C12H8O4 | 5-methoxypsoralens | 16.2 | 102647.6 | 352540.6 | 85.3 |
| Rutin | C27H30O16 | Flavonoid-3-O-glycosides | 6.8 | 94890.1 | 344434.0 | 86.6 |
| Benzyl 6-O-beta-D-glucopyranosyl-beta-D-glucopyranoside | C19H28O11 | O-glycosyl compounds | 5.6 | 105960.8 | 322440.4 | 81.7 |
| (-)-Riboflavin | C17H20N4O6 | Flavins | 6.0 | 103334.3 | 300726.5 | 90.5 |
| Adenosine 5'-monophosphate | C10H14N5O7P | Purine ribonucleoside monophosphates | 2.5 | 97559.9 | 283077.2 | 83.0 |
| 2-acetoxy-4-pentadecylbenzoic acid | C24H38O4 | Acylsalicylic acids | 28.0 | 65244.8 | 279693.8 | 85.0 |
| 3-Buten-2-one, 4-[4-(beta-D-glucopyranosyloxy)-2-hydroxy-2,6,6-trimethylcyclohexylidene]- | C19H30O8 | Terpene glycosides | 9.1 | 61396.9 | 278632.0 | 83.3 |
| PYRIDOXINE | C8H11NO3 | Pyridoxines | 2.5 | 79629.2 | 270769.3 | 90.0 |
| Formononetin | C16H12O4 | 4'-O-methylisoflavones | 16.3 | 77920.1 | 258514.3 | 92.3 |
| Emodin | C15H10O5 | Hydroxyanthraquinones | 10.1 | 45987.9 | 229916.8 | 82.3 |
| Isonicotinic acid | C6H5NO2 | Pyridinecarboxylic acids | 2.2 | 65037.8 | 222132.4 | 83.7 |
| Ala-Leu | C9H18N2O3 | Peptides | 4.0 | 52249.5 | 214115.6 | 83.4 |
| Petunidin 3-galactoside | C22H23O12 | Anthocyanidin-3-O-glycosides | 9.9 | 37455.9 | 180682.0 | 82.9 |
| 2-Butanone, 4-[3-(beta-D-glucopyranosyloxy)-4-hydroxy-2,6,6-trimethyl-1-cyclohexen-1-yl]- | C19H32O8 | Terpene glycosides | 10.7 | 33092.6 | 179217.0 | 81.4 |
| Malvidin-3-O-beta-D-galactoside | C23H25O12 | Anthocyanidin-3-O-glycosides | 9.2 | 39351.6 | 173274.6 | 86.7 |
| Adenosine 3'-monophosphate | C10H14N5O7P | Ribonucleoside 3'-phosphates | 2.2 | 54064.6 | 155573.7 | 80.3 |
| 2-Phenylethyl 2-O-[(2S,3R,4R)-3,4-dihydroxy-4-(hydroxymethyl)tetrahydro-2-furanyl]-beta-D-glucopyranoside | C19H28O10 | O-glycosyl compounds | 6.6 | 38847.9 | 152751.1 | 80.1 |
| Dronedarone | C31H44N2O5S | Aryl-phenylketones | 13.7 | 23386.5 | 143128.3 | 82.4 |
| Wogonin | C16H12O5 | 8-O-methylated flavonoids | 14.8 | 40543.9 | 131818.2 | 80.4 |
| Petunidin-3-O-beta-glucopyranoside | C22H23O12 | Anthocyanidin-3-O-glycosides | 7.5 | 33158.7 | 131211.3 | 82.8 |
| Carbendazim | C9H9N3O2 | 2-benzimidazolylcarbamic acid esters | 6.3 | 33889.6 | 128779.8 | 87.3 |
| Naringenin | C15H12O5 | Flavanones | 6.9 | 28810.3 | 128201.7 | 84.5 |
| sn-Glycero-3-phosphocholine | C8H21NO6P | Glycerophosphocholines | 1.2 | 39632.9 | 125935.5 | 86.7 |
| Guanosine 5'-monophosphate | C10H14N5O8P | Purine ribonucleoside monophosphates | 2.8 | 38348.0 | 123349.2 | 83.0 |
| Oxazepam | C15H11ClN2O2 | 1,4-benzodiazepines | 10.1 | 26048.4 | 123088.1 | 83.7 |
| D-(+)-Glucosamine hydrochloride | C6H13NO5 | Hexoses | 4.4 | 24250.4 | 118075.1 | 90.5 |
| Cytosine | C4H5N3O | Pyrimidones | 1.4 | 23107.3 | 115139.5 | 82.7 |
| 6-Hydroxynicotinic acid | C6H5NO3 | Pyridinecarboxylic acids | 2.7 | 34633.2 | 106877.1 | 80.6 |
| 5'-S-Methylthioadenosine | C11H15N5O3S | 5'-deoxy-5'-thionucleosides | 5.7 | 36185.8 | 105123.9 | 82.3 |
| Biochanin A | C16H12O5 | 4'-O-methylisoflavones | 13.7 | 15067.4 | 95052.9 | 83.0 |
| Oxypurinol | C5H4N4O2 | Xanthines | 2.4 | 28422.4 | 81197.4 | 84.0 |
| Catechin | C15H14O6 | Catechins | 5.7 | 24924.3 | 79921.9 | 87.8 |
| Daidzein | C15H10O4 | Isoflavones | 10.8 | 13694.8 | 79641.5 | 81.3 |
| L-Tyrosine | C9H11NO3 | Tyrosine and derivatives | 2.2 | 23688.2 | 78618.4 | 89.0 |
| Amitryptiline | C20H23N | Dibenzocycloheptenes | 5.0 | 24831.8 | 76842.9 | 80.8 |
| GAMMA-TERPINENE | C10H16 | Branched unsaturated hydrocarbons | 9.4 | 12217.9 | 67975.2 | 81.3 |
| L-(-)-Phenylalanine | C9H11NO2 | Phenylalanine and derivatives | 4.4 | 14756.8 | 66529.6 | 89.3 |
| 4-Pyridoxic acid | C8H9NO4 | Pyridinecarboxylic acids | 3.0 | 16113.3 | 64793.9 | 83.1 |
| Cyanidin-3-glucoside | C21H21O11 | Anthocyanidin-3-O-glycosides | 8.8 | 13809.0 | 61676.0 | 82.8 |
| isorhamnetin-3-O-glucoside | C22H22O12 | Flavonoid-3-O-glycosides | 8.5 | 14262.6 | 61592.2 | 81.2 |
| Atrazine | C8H14ClN5 | Chloro-s-triazines | 5.2 | 11282.7 | 47566.4 | 81.8 |
| 4-Hydroxymandelonitrile | C8H7NO2 | 1-hydroxy-2-unsubstituted benzenoids | 2.7 | 13706.6 | 45223.4 | 85.3 |
| DL-Coniine | C8H17N | Alkaloids and derivatives | 16.3 | 10873.6 | 39910.1 | 92.4 |
| Kynurenic acid | C10H7NO3 | Quinoline carboxylic acids | 5.2 | 12974.4 | 39773.8 | 85.5 |
| beta-D-Glucopyranoside, 3-hydroxy-2-(4-hydroxy-3-methoxyphenyl)propyl | C16H24O9 | O-glycosyl compounds | 4.5 | 10385.3 | 38611.6 | 80.1 |
| (6,6-Dimethylbicyclo[3.1.1]hept-2-yl)methyl 6-O-[(2R,3R,4R)-3,4-dihydroxy-4-(hydroxymethyl)tetrahydro-2-furanyl]-beta-D-glucopyranoside | C21H36O10 | Terpene glycosides | 13.9 | 4150.1 | 24294.2 | 80.8 |
| Coumarin | C9H6O2 | Coumarins and derivatives | 5.4 | 7992.5 | 24089.9 | 80.2 |
| isorhamnetin | C16H12O7 | Flavonols | 8.5 | 4453.3 | 20950.5 | 86.0 |
| Benzimidazole | C7H6N2 | Benzimidazoles | 3.9 | 4648.8 | 17782.0 | 80.4 |
| 2-QUINOLINECARBOXYLIC ACID | C10H7NO2 | Quinoline carboxylic acids | 2.8 | 5195.6 | 16223.6 | 85.0 |

1. TMDCD chemical composition - LC-MS negative ion mode

| Table S3 TMDCD chemical composition - LC-MS negative ion mode | | | | | | |
| --- | --- | --- | --- | --- | --- | --- |
| Component | Molecular formula | Ontology | RT  (min) | Height | Area | Total score |
| [(6-O-Hexopyranosylhexopyranosyl)oxy](phenyl)acetonitrile | C20H27NO11 | Cyanogenic glycosides | 5.8 | 3185303.5 | 15336961.0 | 80.6 |
| Liquiritin | C21H22O9 | Flavonoid O-glycosides | 7.3 | 1903364.8 | 9080694.0 | 84.4 |
| 2-[4-[3-[3,4-dihydroxy-4-(hydroxymethyl)oxolan-2-yl]oxy-4,5-dihydroxy-6-(hydroxymethyl)oxan-2-yl]oxyphenyl]-7-hydroxy-2,3-dihydrochromen-4-one | C26H30O13 | Flavonoid O-glycosides | 7.1 | 1520265.8 | 6655192.5 | 84.8 |
| Cucurbitacin I | C30H42O7 | Cucurbitacins | 15.8 | 1607504.0 | 6169895.0 | 85.1 |
| CITRATE | C6H8O7 | Tricarboxylic acids and derivatives | 1.3 | 930537.9 | 5052217.5 | 90.5 |
| E-3,4,5'-Trihydroxy-3'-glucopyranosylstilbene | C20H22O9 | Stilbene glycosides | 7.3 | 1223474.3 | 4951380.0 | 81.3 |
| Amygdalin | C20H27NO11 | Cyanogenic glycosides | 5.8 | 950935.5 | 4395210.5 | 86.1 |
| Flavanone + 2O, O-Hex | C21H22O9 | Flavonoid O-glycosides | 11.2 | 729680.3 | 4262575.5 | 89.3 |
| Pelargonidin-3-O-glucoside | C21H21O10 | Anthocyanidin-3-O-glycosides | 14.8 | 1005105.0 | 3673683.5 | 85.1 |
| FA 18:1+3O | C18H34O5 | Long-chain fatty acids | 16.0 | 717205.4 | 3506295.3 | 81.3 |
| (2S)-7-[(2S,3R,4S,5S,6R)-4,5-dihydroxy-6-(hydroxymethyl)-3-[(2S,3R,4R,5R,6S)-3,4,5-trihydroxy-6-methyloxan-2-yl]oxyoxan-2-yl]oxy-2-(3,4-dihydroxyphenyl)-5-hydroxy-2,3-dihydrochromen-4-one | C27H32O15 | Flavonoid-7-O-glycosides | 6.6 | 798703.1 | 2976849.5 | 86.1 |
| Glycyrrhizin | C42H62O16 | Triterpene saponins | 15.7 | 896121.3 | 2946980.0 | 89.8 |
| Caffeoyl quinic acid | C16H18O9 | Quinic acids and derivatives | 4.7 | 466358.3 | 2603313.0 | 88.0 |
| Formononetin-7-O-glucoside | C22H22O9 | Isoflavonoid O-glycosides | 11.8 | 410259.6 | 2376622.3 | 87.9 |
| D-(+)-Malic acid | C4H6O5 | Beta hydroxy acids and derivatives | 1.2 | 216680.1 | 2323729.8 | 82.9 |
| Hydroxyferulic acid | C10H10O5 | Hydroxycinnamic acids | 4.4 | 254775.2 | 1807305.9 | 85.4 |
| (3R,5S)-4-[(E)-3-(3,4-dihydroxyphenyl)prop-2-enoyl]oxy-1,3,5-trihydroxycyclohexane-1-carboxylic acid | C16H18O9 | Quinic acids and derivatives | 5.4 | 535354.9 | 1709238.3 | 86.2 |
| 5-Benzofuranpropanoic acid, 4-(beta-D-glucopyranosyloxy)-6-methoxy- | C18H22O10 | Phenolic glycosides | 8.0 | 288622.6 | 1633461.8 | 80.6 |
| Emodin | C15H10O5 | Hydroxyanthraquinones | 19.1 | 327608.3 | 1511777.0 | 93.0 |
| Protocatechuic aldehyde | C7H6O3 | Hydroxybenzaldehydes | 5.6 | 371233.8 | 1417194.3 | 89.1 |
| isoliquiritigenin | C15H12O4 | 2'-Hydroxychalcones | 12.6 | 189136.4 | 1309041.6 | 89.4 |
| Flavanone + 3O, O-Hex | C21H22O10 | Flavonoid-7-O-glycosides | 9.3 | 254860.0 | 1277130.6 | 93.6 |
| 2-Isopropylmalic acid | C7H12O5 | Hydroxy fatty acids | 5.2 | 304477.6 | 1219735.0 | 89.7 |
| Licoricesaponin G2 | C42H62O17 | Triterpene saponins | 15.5 | 355647.1 | 1161961.0 | 84.8 |
| L-(-)-Phenylalanine | C9H11NO2 | Phenylalanine and derivatives | 3.1 | 177153.3 | 985963.0 | 92.2 |
| Cosmosiin | C21H20O10 | Flavonoid-7-O-glycosides | 14.8 | 346843.8 | 979014.2 | 80.8 |
| Benzoic acid | C7H6O2 | Benzoic acids | 6.5 | 244305.8 | 860238.8 | 89.3 |
| Gallic acid | C7H6O5 | Gallic acids | 2.2 | 143017.5 | 788362.2 | 84.7 |
| Stearic acid | C18H36O2 | Long-chain fatty acids | 28.3 | 120899.2 | 731307.5 | 81.9 |
| P-Coumaric acid | C9H8O3 | Hydroxycinnamic acids | 6.9 | 169504.8 | 644082.0 | 88.0 |
| C16H20O10 | C16H20O10 | NA | 6.2 | 190330.8 | 586599.4 | 83.3 |
| Protocatechuic acid | C7H6O4 | Hydroxybenzoic acid derivatives | 4.4 | 93787.9 | 586509.9 | 90.9 |
| Itaconic acid (Not validated) | C5H6O4 | Branched fatty acids | 2.0 | 153908.4 | 569854.8 | 82.9 |
| 5,7-dihydroxy-2-(4-hydroxyphenyl)-8-[3,4,5-trihydroxy-6-(hydroxymethyl)oxan-2-yl]-6-(3,4,5-trihydroxyoxan-2-yl)chromen-4-one | C26H28O14 | Flavonoid 8-C-glycosides | 6.1 | 179075.8 | 551463.0 | 82.7 |
| Guanosine | C10H13N5O5 | Purine nucleosides | 1.9 | 136990.5 | 510280.9 | 89.0 |
| Petunidin-3-O-beta-glucopyranoside | C22H23O12 | Anthocyanidin-3-O-glycosides | 7.8 | 128269.1 | 495239.3 | 85.6 |
| SUCCINIC ACID | C4H6O4 | Dicarboxylic acids and derivatives | 1.9 | 129201.8 | 493727.8 | 90.5 |
| Itaconic acid | C5H6O4 | Branched fatty acids | 3.1 | 48596.7 | 484419.1 | 85.5 |
| Tryptophan | C11H12N2O2 | Indolyl carboxylic acids and derivatives | 4.9 | 135321.4 | 478025.9 | 89.1 |
| Quercetin-3-O-rutinoside | C27H30O16 | Flavonoid-3-O-glycosides | 6.8 | 118842.3 | 430260.7 | 86.6 |
| Salicylic acid | C7H6O3 | Salicylic acids | 7.1 | 72592.5 | 427115.5 | 85.7 |
| Apigenin-8-C-glucoside-2'-rhamnoside | C27H30O14 | Flavonoid 8-C-glycosides | 6.6 | 99617.6 | 410345.6 | 80.6 |
| AcetylPhenylalanine | C11H13NO3 | Phenylalanine and derivatives | 6.2 | 121384.0 | 409956.8 | 83.5 |
| 3,4-DIHYDROXYBENZOIC ACID | C7H6O4 | Hydroxybenzoic acid derivatives | 3.7 | 58480.9 | 404507.6 | 87.4 |
| 3-Hydroxy-3-methylglutaric acid | C6H10O5 | Hydroxy fatty acids | 1.9 | 119123.6 | 397708.2 | 84.9 |
| 4-hydroxy-7-methyl-2-[2-[3,4,5-trihydroxy-6-(hydroxymethyl)oxan-2-yl]oxypropan-2-yl]-2,3-dihydrofuro[3,2-g]chromen-5-one | C21H26O10 | Furanochromones | 11.5 | 71264.3 | 364567.9 | 82.2 |
| L-5-Oxoproline | C5H7NO3 | Alpha amino acids and derivatives | 1.4 | 53130.1 | 363267.3 | 85.8 |
| Sebacic acid | C10H18O4 | Medium-chain fatty acids | 13.7 | 53470.1 | 339892.1 | 88.4 |
| Benzoic acid + 2O, O-Hex | C13H16O9 | Phenolic glycosides | 2.3 | 73189.4 | 323709.0 | 88.8 |
| Biochanin-7-O-glucoside | C22H22O10 | Isoflavonoid O-glycosides | 14.9 | 81484.9 | 278098.7 | 80.1 |
| Palmitic acid | C16H32O2 | Long-chain fatty acids | 26.7 | 54920.8 | 266666.5 | 83.5 |
| C21H32O10 | C21H32O10 | NA | 5.4 | 84697.1 | 254699.8 | 83.9 |
| Licoricesaponin H2 | C42H62O16 | Triterpene saponins | 14.6 | 31751.8 | 246059.9 | 84.2 |
| Ferulic acid | C10H10O4 | Hydroxycinnamic acids | 7.5 | 54331.4 | 230235.1 | 82.2 |
| Adenosine 3':5'-cyclicmonophosphate | C10H12N5O6P | 3',5'-cyclic purine nucleotides | 1.8 | 64517.9 | 224397.2 | 81.7 |
| (6,6-Dimethylbicyclo[3.1.1]hept-2-yl)methyl 6-O-[(2R,3R,4R)-3,4-dihydroxy-4-(hydroxymethyl)tetrahydro-2-furanyl]-beta-D-glucopyranoside | C21H36O10 | Terpene glycosides | 13.6 | 38084.3 | 222021.4 | 84.1 |
| Ala-Ile | C9H18N2O3 | Dipeptides | 4.1 | 34538.5 | 191545.4 | 92.4 |
| Benzoic acid + 2O, O-Pen | C12H14O8 | p-Hydroxybenzoic acid alkyl esters | 3.9 | 30360.2 | 189793.7 | 85.3 |
| C8H8O3 | C8H8O3 | NA | 5.2 | 44568.7 | 184881.3 | 82.9 |
| Isoquercetrin | C21H20O12 | Flavonoid-3-O-glycosides | 11.0 | 34356.6 | 184629.6 | 89.3 |
| L-Leucine | C6H13NO2 | Leucine and derivatives | 1.8 | 55267.6 | 183448.2 | 86.1 |
| Hydroxysebacic acid | C10H18O5 | Medium-chain hydroxy acids and derivatives | 7.4 | 48900.4 | 177063.4 | 83.2 |
| Eleutheroside E | C34H46O18 | Lignan glycosides | 6.2 | 55431.3 | 175209.9 | 83.0 |
| MANDELIC ACID | C8H8O3 | Benzene and substituted derivatives | 6.7 | 32643.6 | 162872.3 | 86.9 |
| Coumaroyl Hexoside | C15H18O8 | Hydroxycinnamic acid glycosides | 6.4 | 41112.3 | 154935.6 | 81.5 |
| Isofraxidin | C11H10O5 | 7-hydroxycoumarins | 7.8 | 37341.0 | 151190.9 | 86.8 |
| Naringenin | C15H12O5 | Flavanones | 15.3 | 44631.7 | 148264.0 | 88.7 |
| Syringic acid | C9H10O5 | Gallic acid and derivatives | 6.1 | 45036.5 | 141350.5 | 80.1 |
| Malonyltryptophan | C14H14N2O5 | N-acyl-alpha amino acids | 6.9 | 36336.4 | 140025.2 | 82.6 |
| Vitexin | C21H20O10 | Flavonoid 8-C-glycosides | 7.0 | 37263.1 | 134687.4 | 84.8 |
| Scopoletin | C10H8O4 | 7-hydroxycoumarins | 7.5 | 30354.0 | 128897.4 | 90.4 |
| Caffeic acid | C9H8O4 | Hydroxycinnamic acids | 5.9 | 41309.2 | 125461.0 | 90.4 |
| Coniferyl aldehyde | C10H10O3 | Methoxyphenols | 9.2 | 24394.5 | 125190.3 | 90.7 |
| 5,7-dihydroxy-2-[4-hydroxy-3-[(2S,3R,4S,5S,6R)-3,4,5-trihydroxy-6-(hydroxymethyl)oxan-2-yl]oxyphenyl]-3-methoxychromen-4-one | C22H22O12 | Flavonoid O-glycosides | 10.0 | 24242.9 | 122636.1 | 81.0 |
| 2-Hydroxyphenylacetic acid | C8H8O3 | 2(hydroxyphenyl)acetic acids | 10.0 | 13339.6 | 122029.7 | 85.2 |
| Syringaldehyde | C9H10O4 | Methoxyphenols | 7.4 | 31057.9 | 117400.6 | 85.4 |
| Gly-Leu | C8H16N2O3 | Peptides | 4.0 | 16714.1 | 103441.5 | 86.2 |
| 4-Nitrophenol | C6H5NO3 | Nitrophenols | 10.5 | 15048.9 | 99130.0 | 90.6 |
| Hydroxysuberic acid | C8H14O5 | Medium-chain hydroxy acids and derivatives | 6.2 | 30266.4 | 98271.1 | 84.3 |
| Formononetin | C16H12O4 | 4'-O-methylisoflavones | 16.3 | 29553.6 | 96425.6 | 83.5 |
| Vanillic acid | C8H8O4 | M-methoxybenzoic acids and derivatives | 6.0 | 29750.9 | 89179.0 | 91.7 |
| Isorhamnetin-3-glucoside | C22H22O12 | Flavonoid-3-O-glycosides | 8.5 | 19028.4 | 87366.5 | 83.7 |
| C6H12O4 | C6H12O4 | NA | 2.1 | 16117.5 | 83829.5 | 86.9 |
| Pantothenate | C9H17NO5 | Secondary alcohols | 4.0 | 15515.8 | 82055.2 | 84.8 |
| Kaempferol-7-O-glucoside | C21H20O11 | Flavonoid-7-O-glycosides | 12.0 | 13956.8 | 81472.9 | 80.2 |
| Salicylic alcohol | C7H8O2 | Benzyl alcohols | 7.7 | 18142.9 | 78218.0 | 81.7 |
| Adenosine 3'-monophosphate | C10H14N5O7P | Ribonucleoside 3'-phosphates | 2.0 | 16566.2 | 76407.8 | 82.8 |
| Inosine | C10H12N4O5 | Purine nucleosides | 1.9 | 20877.7 | 76093.9 | 88.7 |
| 5-O-Caffeoylquinic acid methyl ester | C17H20O9 | Quinic acids and derivatives | 6.8 | 11827.8 | 65729.6 | 82.2 |
| Chrysin | C15H10O4 | Flavones | 10.9 | 10503.4 | 64470.8 | 84.8 |
| trans-Cinnamic acid | C9H8O2 | Cinnamic acids | 3.1 | 11622.3 | 63483.8 | 83.9 |
| Methylsuccinic acid | C5H8O4 | Methyl-branched fatty acids | 2.7 | 10402.8 | 63065.7 | 93.2 |
| Sinapic acid | C11H12O5 | Hydroxycinnamic acids | 7.4 | 15775.7 | 61969.6 | 86.9 |
| C12-AS (TENTATIVE) | C12H26O4S | Sulfuric acid monoesters | 18.8 | 17623.5 | 61827.9 | 92.1 |
| Acetylleucine | C8H15NO3 | Leucine and derivatives | 6.0 | 21139.5 | 60419.1 | 86.5 |
| DL-p-Hydroxyphenyllactic acid | C9H10O4 | Phenylpropanoic acids | 4.9 | 16345.1 | 59939.2 | 92.5 |
| Ethylparaben | C9H10O3 | p-Hydroxybenzoic acid alkyl esters | 8.1 | 13639.6 | 58653.8 | 85.4 |
| 7-hydroxy-coumarin | C9H6O3 | 7-hydroxycoumarins | 8.1 | 13547.8 | 58497.9 | 86.5 |
| 5-hydroxy-2-(4-hydroxyphenyl)-7-[(2S,3R,4S,5S,6R)-3,4,5-trihydroxy-6-(hydroxymethyl)oxan-2-yl]oxychromen-4-one | C21H20O10 | Flavonoid-7-O-glycosides | 8.8 | 11537.4 | 55594.2 | 88.9 |
| Luteolin-7-O-glucoside | C21H20O11 | Flavonoid-7-O-glycosides | 15.5 | 17507.4 | 52981.8 | 82.1 |
| Acetylleucine | C8H15NO3 | Leucine and derivatives | 6.5 | 15131.7 | 48520.4 | 80.2 |
| (E)-3-[2-[(2S,3R,4S,5S,6R)-3,4,5-trihydroxy-6-(hydroxymethyl)oxan-2-yl]oxyphenyl]prop-2-enoic acid | C15H18O8 | Phenolic glycosides | 5.0 | 10677.6 | 48453.8 | 88.7 |
| 3-Methyladipic acid | C7H12O4 | Medium-chain fatty acids | 6.2 | 13530.3 | 42755.5 | 85.0 |
| 3-Caffeoylquinic acid | C16H18O9 | Quinic acids and derivatives | 4.0 | 6193.8 | 39409.4 | 88.1 |
| Uric acid | C5H4N4O3 | Xanthines | 1.4 | 8618.0 | 36116.1 | 80.1 |
| cis-Aconitate | C6H6O6 | Tricarboxylic acids and derivatives | 8.3 | 7658.5 | 35880.0 | 83.7 |
| Lactic acid | C3H6O3 | Alpha hydroxy acids and derivatives | 1.4 | 6067.9 | 35417.0 | 82.9 |
| Flavonol base + 4O, O-dHex | C21H20O11 | Flavonoid-3-O-glycosides | 8.4 | 6666.9 | 33997.7 | 86.3 |
| L-(+)-Arginine | C6H14N4O2 | L-alpha-amino acids | 1.2 | 8946.3 | 33992.2 | 83.9 |
| Homogenentisic acid | C8H8O4 | 2(hydroxyphenyl)acetic acids | 6.6 | 7661.8 | 33257.2 | 82.4 |
| Mitragynine | C23H30N2O4 | Corynanthean-type alkaloids | 16.0 | 7185.9 | 32616.3 | 89.0 |
| Apigenin-6-C-glucoside-7-O-glucoside | C27H30O15 | Flavonoid-7-O-glycosides | 11.8 | 5654.0 | 32255.1 | 80.7 |
| Feruloyl Lactate | C13H14O7 | NA | 5.7 | 9396.2 | 32152.7 | 81.6 |
| Pimelic acid | C7H12O4 | Medium-chain fatty acids | 5.9 | 10683.4 | 30066.9 | 82.6 |
| 3-Hydroxycinnamic acid | C9H8O3 | Hydroxycinnamic acids | 9.5 | 5597.3 | 28635.9 | 87.6 |
| Adenine | C5H5N5 | 6-aminopurines | 1.6 | 7793.4 | 26886.7 | 89.9 |
| Arctigenin | C21H24O6 | Dibenzylbutyrolactone lignans | 11.3 | 4646.4 | 24977.2 | 81.0 |
| 4-Methoxycinnamic acid | C10H10O3 | Cinnamic acids | 14.8 | 6221.8 | 24749.5 | 89.3 |
| (1R,2S,5aR,5bR,7aS,10R,12bR)-2-Hydroxy-10-isopropenyl-3,3,5a,5b,12b-pentamethyloctadecahydrodicyclopenta[a,i]phenanthrene-1,7a(1H)-dicarboxylic acid | C30H46O5 | Triterpenoids | 17.9 | 3555.4 | 23712.4 | 80.1 |
| gamma-Glutamylleucine | C11H20N2O5 | Dipeptides | 5.0 | 8345.8 | 23347.4 | 83.8 |
| Cyanidin | C15H11O6 | 7-hydroxyflavonoids | 16.9 | 5076.6 | 22491.8 | 81.4 |
| Dihydrokaempferol | C15H12O6 | Flavanonols | 9.7 | 3930.0 | 21759.9 | 82.1 |
| Vanillin | C8H8O3 | Methoxyphenols | 7.6 | 5209.1 | 21718.5 | 82.0 |
| Dodecylbenzenesulfonic acid | C18H30O3S | Benzenesulfonic acids and derivatives | 20.9 | 4767.4 | 19725.0 | 83.2 |
| 3'-O-Methylguanosine | C11H15N5O5 | Purine nucleosides | 3.0 | 3461.6 | 17402.8 | 82.7 |
| Heptadecanoic acid | C17H34O2 | Long-chain fatty acids | 27.2 | 2313.2 | 17294.8 | 81.5 |
| DL-4-Hydroxy-3-methoxymandelic acid | C9H10O5 | Methoxyphenols | 2.3 | 2593.0 | 16348.8 | 89.6 |
| 4-Hydroxyquinoline | C9H7NO | Hydroquinolones | 3.2 | 2658.4 | 16119.4 | 91.1 |
| 9Z,12Z-Linoleic acid | C18H32O2 | Lineolic acids and derivatives | 29.3 | 3055.9 | 14060.6 | 84.3 |
| Bufalin | C24H34O4 | Bufanolides and derivatives | 15.2 | 4087.7 | 13912.2 | 80.5 |
| 9-Trans-Palmitelaidic acid | C16H30O2 | Long-chain fatty acids | 25.3 | 1583.3 | 7560.4 | 83.0 |
| Myristic acid | C14H28O2 | Long-chain fatty acids | 24.8 | 1646.0 | 6931.6 | 85.7 |
| Pseudojervine | C33H49NO8 | Steroidal saponins | 12.0 | 1242.8 | 6520.9 | 87.1 |
| Pygenic acid B b | C30H48O5 | Triterpenoids | 19.3 | 1419.0 | 5193.2 | 82.0 |

1. AHR test

| Table S4 AHR test | | | | |
| --- | --- | --- | --- | --- |
| index | Methacholine (mg/ml) | group | mean | SEM |
| Penh | 0 | NC | 0.74 | 0.11 |
|  |  | OVA | 0.68 | 0.02 |
|  |  | OVA+Dex | 0.69 | 0.07 |
|  |  | OVA+TMDCD | 0.79 | 0.04 |
|  | 3.125 | NC | 0.82 | 0.11 |
|  |  | OVA | 2.87 | 0.72 |
|  |  | OVA+Dex | 1.43 | 0.37 |
|  |  | OVA+TMDCD | 1.34 | 0.38 |
|  | 6.25 | NC | 1.34 | 0.19 |
|  |  | OVA | 3.69 | 0.62 |
|  |  | OVA+Dex | 2.76 | 0.55 |
|  |  | OVA+TMDCD | 1.87 | 0.49 |
|  | 12.5 | NC | 2.45 | 0.22 |
|  |  | OVA | 7.80 | 2.04 |
|  |  | OVA+Dex | 4.18 | 0.65 |
|  |  | OVA+TMDCD | 3.66 | 0.67 |
|  | 25 | NC | 4.14 | 0.32 |
|  |  | OVA | 10.12 | 1.65 |
|  |  | OVA+Dex | 5.24 | 0.57 |
|  |  | OVA+TMDCD | 5.25 | 0.64 |
| Pause | 0 | NC | 0.86 | 0.07 |
|  |  | OVA | 0.94 | 0.04 |
|  |  | OVA+Dex | 1.03 | 0.14 |
|  |  | OVA+TMDCD | 1.01 | 0.03 |
|  | 3.125 | NC | 0.95 | 0.07 |
|  |  | OVA | 2.33 | 0.32 |
|  |  | OVA+Dex | 1.44 | 0.15 |
|  |  | OVA+TMDCD | 1.34 | 0.22 |
|  | 6.25 | NC | 1.33 | 0.13 |
|  |  | OVA | 2.96 | 0.35 |
|  |  | OVA+Dex | 2.25 | 0.33 |
|  |  | OVA+TMDCD | 1.80 | 0.34 |
|  | 12.5 | NC | 2.12 | 0.16 |
|  |  | OVA | 5.16 | 1.02 |
|  |  | OVA+Dex | 3.16 | 0.44 |
|  |  | OVA+TMDCD | 2.81 | 0.40 |
|  | 25 | NC | 2.96 | 0.19 |
|  |  | OVA | 6.43 | 0.73 |
|  |  | OVA+Dex | 3.78 | 0.36 |
|  |  | OVA+TMDCD | 3.57 | 0.34 |
| R_L_ | 0 | NC | 1.14 | 0.04 |
|  |  | OVA | 1.59 | 0.25 |
|  |  | OVA+Dex | 1.40 | 0.07 |
|  |  | OVA+TMDCD | 1.19 | 0.04 |
|  | 3.125 | NC | 1.46 | 0.04 |
|  |  | OVA | 2.72 | 0.06 |
|  |  | OVA+Dex | 2.40 | 0.08 |
|  |  | OVA+TMDCD | 1.91 | 0.01 |
|  | 6.25 | NC | 2.24 | 0.11 |
|  |  | OVA | 5.49 | 0.04 |
|  |  | OVA+Dex | 3.67 | 0.31 |
|  |  | OVA+TMDCD | 3.24 | 0.10 |
|  | 12.5 | NC | 3.16 | 0.07 |
|  |  | OVA | 6.31 | 0.18 |
|  |  | OVA+Dex | 4.41 | 0.32 |
|  |  | OVA+TMDCD | 4.24 | 0.12 |
| Cdyn | 0 | NC | 0.021 | 0.001 |
|  |  | OVA | 0.014 | 0.003 |
|  |  | OVA+Dex | 0.018 | 0.002 |
|  |  | OVA+TMDCD | 0.021 | 0.001 |
|  | 3.125 | NC | 0.019 | 0.001 |
|  |  | OVA | 0.010 | 0.000 |
|  |  | OVA+Dex | 0.013 | 0.002 |
|  |  | OVA+TMDCD | 0.016 | 0.001 |
|  | 6.25 | NC | 0.011 | 0.001 |
|  |  | OVA | 0.006 | 0.001 |
|  |  | OVA+Dex | 0.007 | 0.001 |
|  |  | OVA+TMDCD | 0.007 | 0.000 |
|  | 12.5 | NC | 0.007 | 0.000 |
|  |  | OVA | 0.004 | 0.000 |
|  |  | OVA+Dex | 0.003 | 0.001 |
|  |  | OVA+TMDCD | 0.005 | 0.000 |

1. Eosinophils count and IgE level in BALF.

| Table S5 Eosinophils count and IgE level in BALF | | | |
| --- | --- | --- | --- |
| index | group | mean | SEM |
| Eosinophils (10^9^/L) | NC | 0.18 | 0.02 |
|  | OVA | 3.52 | 0.45 |
|  | OVA+Dex | 0.72 | 0.17 |
|  | OVA+TMDCD | 1.38 | 0.38 |
| IgE (ng/ml) | NC | 0.58 | 0.02 |
|  | OVA | 1.02 | 0.19 |
|  | OVA+Dex | 0.63 | 0.03 |
|  | OVA+TMDCD | 0.63 | 0.04 |

1. PAS positive area in lung tissue.

| Table S6 PAS positive area in lung tissue (%) | | |
| --- | --- | --- |
| group | mean | SEM |
| NC | 0.03 | 0.01 |
| OVA | 4.01 | 1.56 |
| OVA+Dex | 1.04 | 0.72 |
| OVA+TMDCD | 1.47 | 0.77 |

1. Total IgE, OVA-specific IgE, Histamine, LTC4 of serum.

| Table S7 Total IgE, OVA-specific IgE, Histamine, LTC4 of serum | | | |
| --- | --- | --- | --- |
| index | group | mean | SEM |
| Total IgE (ng/ml) | NC | 7.19 | 0.16 |
|  | OVA | 28.86 | 2.45 |
|  | OVA+Dex | 16.25 | 1.49 |
|  | OVA+TMDCD | 11.06 | 0.68 |
| OVA-specific IgE (OD) | NC | 0.07 | 0.00 |
|  | OVA | 2.19 | 0.07 |
|  | OVA+Dex | 1.91 | 0.03 |
|  | OVA+TMDCD | 1.92 | 0.09 |
| Histamine (pg/ml) | NC | 771.98 | 36.27 |
|  | OVA | 2562.73 | 193.25 |
|  | OVA+Dex | 1046.26 | 60.40 |
|  | OVA+TMDCD | 1253.70 | 136.74 |
| LTC4 (ng/ml) | NC | 265.90 | 13.24 |
|  | OVA | 807.31 | 26.25 |
|  | OVA+Dex | 462.41 | 15.81 |
|  | OVA+TMDCD | 489.26 | 32.70 |

1. Differentially expressed proteins of serum.

| Table S8 Differentially expressed proteins of serum (pg/ml) | | | |
| --- | --- | --- | --- |
| Protein ID | group | mean | SEM |
| OPG | NC | 4719.78 | 808.07 |
|  | OVA | 32036.30 | 4926.73 |
|  | OVA+Dex | 25495.70 | 11874.96 |
|  | OVA+TMDCD | 18921.28 | 2331.05 |
| Fractalkine | NC | 570.89 | 99.32 |
|  | OVA | 2579.65 | 604.55 |
|  | OVA+Dex | 610.09 | 142.17 |
|  | OVA+TMDCD | 420.00 | 45.50 |
| Tryptase ε | NC | 525.21 | 104.01 |
|  | OVA | 1783.27 | 453.05 |
|  | OVA+Dex | 1145.27 | 134.66 |
|  | OVA+TMDCD | 621.08 | 76.18 |
| IL-25 | NC | 837.20 | 135.53 |
|  | OVA | 2796.08 | 839.73 |
|  | OVA+Dex | 1210.32 | 124.71 |
|  | OVA+TMDCD | 917.10 | 29.56 |
| CCL19 | NC | 442.00 | 40.55 |
|  | OVA | 1226.00 | 299.68 |
|  | OVA+Dex | 714.08 | 75.92 |
|  | OVA+TMDCD | 464.73 | 44.34 |
| MCP-1 | NC | 1665.62 | 89.41 |
|  | OVA | 4614.24 | 744.74 |
|  | OVA+Dex | 4363.08 | 672.05 |
|  | OVA+TMDCD | 1832.38 | 81.88 |
| OX40L | NC | 551.10 | 136.76 |
|  | OVA | 1234.04 | 267.46 |
|  | OVA+Dex | 585.34 | 43.05 |
|  | OVA+TMDCD | 494.81 | 68.28 |
| Axl | NC | 26472.15 | 3581.85 |
|  | OVA | 49317.63 | 5350.69 |
|  | OVA+Dex | 51693.55 | 7949.80 |
|  | OVA+TMDCD | 31922.87 | 5094.57 |
| CCL22 | NC | 13139.22 | 1299.65 |
|  | OVA | 23922.58 | 1463.71 |
|  | OVA+Dex | 22009.21 | 4428.62 |
|  | OVA+TMDCD | 13211.54 | 807.75 |
| CD30 | NC | 2877.06 | 249.42 |
|  | OVA | 5188.20 | 584.76 |
|  | OVA+Dex | 4060.27 | 534.34 |
|  | OVA+TMDCD | 2924.38 | 460.82 |
| G-CSF | NC | 4675.14 | 259.44 |
|  | OVA | 7437.46 | 718.93 |
|  | OVA+Dex | 7869.82 | 1374.29 |
|  | OVA+TMDCD | 2810.66 | 532.24 |
| VEGF | NC | 10257.21 | 622.89 |
|  | OVA | 16228.69 | 2062.69 |
|  | OVA+Dex | 13088.51 | 400.06 |
|  | OVA+TMDCD | 12685.18 | 1990.27 |
| E-selectin | NC | 119898.69 | 5187.14 |
|  | OVA | 177912.82 | 8862.30 |
|  | OVA+Dex | 162413.68 | 8235.51 |
|  | OVA+TMDCD | 122589.33 | 6288.35 |
| OPN | NC | 143493.38 | 2066.46 |
|  | OVA | 199051.49 | 6373.69 |
|  | OVA+Dex | 179832.68 | 10100.61 |
|  | OVA+TMDCD | 152770.06 | 8696.08 |
| CCL5 | NC | 3839.07 | 562.15 |
|  | OVA | 4995.41 | 343.99 |
|  | OVA+Dex | 4240.49 | 234.42 |
|  | OVA+TMDCD | 3508.92 | 254.08 |
| P-selectin | NC | 101697.02 | 2837.81 |
|  | OVA | 131174.66 | 6546.71 |
|  | OVA+Dex | 114622.51 | 8068.71 |
|  | OVA+TMDCD | 100176.44 | 3368.78 |
| Gas6 | NC | 22571.99 | 1909.80 |
|  | OVA | 28390.79 | 1196.45 |
|  | OVA+Dex | 26743.65 | 1588.60 |
|  | OVA+TMDCD | 22653.73 | 1576.50 |
| TSLP | NC | 3476.43 | 154.88 |
|  | OVA | 4333.67 | 266.56 |
|  | OVA+Dex | 4032.37 | 162.06 |
|  | OVA+TMDCD | 3202.59 | 176.46 |

1. GO (CC) enrichment analysis of differentially expressed protein

| Table S9 GO (CC) enrichment analysis of differentially expressed protein | | | | |
| --- | --- | --- | --- | --- |
| term ID | Term Description | Gene  Counts | Background  Gene Count | *P* |
| GO:0005615 | extracellular space | 14 | 1131 | 0.000 |
| GO:0005576 | extracellular region | 15 | 2044 | 0.000 |
| GO:0009986 | cell surface | 6 | 796 | 0.001 |
| GO:0031226 | intrinsic component of plasma membrane | 5 | 1183 | 0.045 |
| GO:0048471 | perinuclear region of cytoplasm | 4 | 730 | 0.045 |

1. GO (MF) enrichment analysis of differentially expressed protein

| Table S10 GO (MF) enrichment analysis of differentially expressed protein | | | | |
| --- | --- | --- | --- | --- |
| term ID | Term Description | Gene  Counts | Background  Gene Count | *P* |
| GO:0005515 | protein binding | 15 | 6456 | 0.000 |
| GO:0048018 | receptor ligand activity | 11 | 429 | 0.000 |
| GO:0005125 | cytokine activity | 10 | 197 | 0.000 |
| GO:0005126 | cytokine receptor binding | 9 | 267 | 0.000 |
| GO:0043168 | anion binding | 7 | 2578 | 0.017 |
| GO:0097367 | carbohydrate derivative binding | 6 | 2051 | 0.024 |
| GO:0048020 | CCR chemokine receptor binding | 5 | 35 | 0.000 |
| GO:0008009 | chemokine activity | 5 | 41 | 0.000 |
| GO:0008201 | heparin binding | 4 | 139 | 0.000 |
| GO:0004888 | transmembrane signaling receptor activity | 4 | 863 | 0.024 |
| GO:0070851 | growth factor receptor binding | 3 | 141 | 0.002 |
| GO:0005509 | calcium ion binding | 3 | 594 | 0.049 |
| GO:0033691 | sialic acid binding | 2 | 8 | 0.000 |
| GO:0070492 | oligosaccharide binding | 2 | 13 | 0.001 |
| GO:0030296 | protein tyrosine kinase activator activity | 2 | 14 | 0.001 |
| GO:0005031 | tumor necrosis factor-activated receptor activity | 2 | 20 | 0.001 |
| GO:0042056 | chemoattractant activity | 2 | 29 | 0.002 |
| GO:0001786 | phosphatidylserine binding | 2 | 41 | 0.004 |
| GO:0050840 | extracellular matrix binding | 2 | 56 | 0.007 |
| GO:0019838 | growth factor binding | 2 | 138 | 0.029 |
| GO:0008083 | growth factor activity | 2 | 146 | 0.031 |

1. GO (BP) enrichment analysis of differentially expressed protein

| Table S11 GO (BP) enrichment analysis of differentially expressed protein | | | | |
| --- | --- | --- | --- | --- |
| term ID | Term Description | Gene  Counts | Background  Gene Count | *P* |
| GO:0002376 | immune system process | 16 | 1703 | 0.000 |
| GO:0050896 | response to stimulus | 16 | 6616 | 0.000 |
| GO:0050794 | regulation of cellular process | 16 | 9045 | 0.000 |
| GO:0010033 | response to organic substance | 15 | 2553 | 0.000 |
| GO:0006950 | response to stress | 15 | 2899 | 0.000 |
| GO:0048522 | positive regulation of cellular process | 15 | 4827 | 0.000 |
| GO:0051716 | cellular response to stimulus | 15 | 5142 | 0.000 |
| GO:0071310 | cellular response to organic substance | 14 | 1858 | 0.000 |
| GO:0051239 | regulation of multicellular organismal process | 14 | 2858 | 0.000 |
| GO:0034097 | response to cytokine | 13 | 792 | 0.000 |
| GO:0006955 | immune response | 13 | 914 | 0.000 |
| GO:0051240 | positive regulation of multicellular organismal process | 13 | 1695 | 0.000 |
| GO:0009605 | response to external stimulus | 13 | 2021 | 0.000 |
| GO:0009966 | regulation of signal transduction | 13 | 2823 | 0.000 |
| GO:0010604 | positive regulation of macromolecule metabolic process | 13 | 3004 | 0.000 |
| GO:0031325 | positive regulation of cellular metabolic process | 13 | 3004 | 0.000 |
| GO:0065009 | regulation of molecular function | 13 | 3081 | 0.000 |
| GO:0048519 | negative regulation of biological process | 13 | 4771 | 0.000 |
| GO:0071345 | cellular response to cytokine stimulus | 12 | 676 | 0.000 |
| GO:0002684 | positive regulation of immune system process | 12 | 771 | 0.000 |
| GO:0040011 | locomotion | 12 | 1065 | 0.000 |
| GO:0006952 | defense response | 12 | 1079 | 0.000 |
| GO:0006928 | movement of cell or subcellular component | 12 | 1223 | 0.000 |
| GO:0010647 | positive regulation of cell communication | 12 | 1608 | 0.000 |
| GO:0023056 | positive regulation of signaling | 12 | 1615 | 0.000 |
| GO:0010941 | regulation of cell death | 12 | 1640 | 0.000 |
| GO:0048584 | positive regulation of response to stimulus | 12 | 1922 | 0.000 |
| GO:0032879 | regulation of localization | 12 | 2579 | 0.000 |
| GO:0051173 | positive regulation of nitrogen compound metabolic process | 12 | 2866 | 0.000 |
| GO:0065008 | regulation of biological quality | 12 | 3420 | 0.000 |
| GO:0007165 | signal transduction | 12 | 3594 | 0.000 |
| GO:0080090 | regulation of primary metabolic process | 12 | 5184 | 0.001 |
| GO:0006954 | inflammatory response | 11 | 454 | 0.000 |
| GO:0010469 | regulation of signaling receptor activity | 11 | 550 | 0.000 |
| GO:0016477 | cell migration | 11 | 758 | 0.000 |
| GO:1902533 | positive regulation of intracellular signal transduction | 11 | 927 | 0.000 |
| GO:0042981 | regulation of apoptotic process | 11 | 1476 | 0.000 |
| GO:0042127 | regulation of cell population proliferation | 11 | 1594 | 0.000 |
| GO:0051049 | regulation of transport | 11 | 1782 | 0.000 |
| GO:2000026 | regulation of multicellular organismal development | 11 | 1961 | 0.000 |
| GO:0051704 | multi-organism process | 11 | 2092 | 0.000 |
| GO:0048731 | system development | 11 | 4072 | 0.000 |
| GO:0048523 | negative regulation of cellular process | 11 | 4336 | 0.001 |
| GO:0050900 | leukocyte migration | 10 | 186 | 0.000 |
| GO:0051050 | positive regulation of transport | 10 | 987 | 0.000 |
| GO:0060548 | negative regulation of cell death | 10 | 1004 | 0.000 |
| GO:0051707 | response to other organism | 10 | 1050 | 0.000 |
| GO:0051247 | positive regulation of protein metabolic process | 10 | 1556 | 0.000 |
| GO:0044093 | positive regulation of molecular function | 10 | 1593 | 0.000 |
| GO:0007166 | cell surface receptor signaling pathway | 10 | 1683 | 0.000 |
| GO:0051128 | regulation of cellular component organization | 10 | 2337 | 0.000 |
| GO:0030154 | cell differentiation | 10 | 3462 | 0.001 |
| GO:0002687 | positive regulation of leukocyte migration | 9 | 143 | 0.000 |
| GO:0006935 | chemotaxis | 9 | 432 | 0.000 |
| GO:0001817 | regulation of cytokine production | 9 | 592 | 0.000 |
| GO:0032101 | regulation of response to external stimulus | 9 | 811 | 0.000 |
| GO:0043066 | negative regulation of apoptotic process | 9 | 884 | 0.000 |
| GO:0008284 | positive regulation of cell population proliferation | 9 | 913 | 0.000 |
| GO:0001934 | positive regulation of protein phosphorylation | 9 | 937 | 0.000 |
| GO:1901700 | response to oxygen-containing compound | 9 | 1429 | 0.000 |
| GO:0045595 | regulation of cell differentiation | 9 | 1751 | 0.000 |
| GO:0010556 | regulation of macromolecule biosynthetic process | 9 | 3417 | 0.003 |
| GO:0016043 | cellular component organization | 9 | 4560 | 0.017 |
| GO:0030595 | leukocyte chemotaxis | 8 | 114 | 0.000 |
| GO:0050867 | positive regulation of cell activation | 8 | 314 | 0.000 |
| GO:0045785 | positive regulation of cell adhesion | 8 | 383 | 0.000 |
| GO:0032103 | positive regulation of response to external stimulus | 8 | 402 | 0.000 |
| GO:0098542 | defense response to other organism | 8 | 735 | 0.000 |
| GO:0033993 | response to lipid | 8 | 825 | 0.000 |
| GO:0032880 | regulation of protein localization | 8 | 947 | 0.000 |
| GO:0045597 | positive regulation of cell differentiation | 8 | 1033 | 0.000 |
| GO:0051336 | regulation of hydrolase activity | 8 | 1128 | 0.000 |
| GO:0080134 | regulation of response to stress | 8 | 1167 | 0.000 |
| GO:0051241 | negative regulation of multicellular organismal process | 8 | 1191 | 0.000 |
| GO:0051130 | positive regulation of cellular component organization | 8 | 1197 | 0.000 |
| GO:0043085 | positive regulation of catalytic activity | 8 | 1239 | 0.000 |
| GO:0031326 | regulation of cellular biosynthetic process | 8 | 3549 | 0.014 |
| GO:0071621 | granulocyte chemotaxis | 7 | 65 | 0.000 |
| GO:0071356 | cellular response to tumor necrosis factor | 7 | 160 | 0.000 |
| GO:0070374 | positive regulation of ERK1 and ERK2 cascade | 7 | 204 | 0.000 |
| GO:0002696 | positive regulation of leukocyte activation | 7 | 300 | 0.000 |
| GO:0019221 | cytokine-mediated signaling pathway | 7 | 313 | 0.000 |
| GO:1903706 | regulation of hemopoiesis | 7 | 377 | 0.000 |
| GO:0001819 | positive regulation of cytokine production | 7 | 383 | 0.000 |
| GO:0045087 | innate immune response | 7 | 534 | 0.000 |
| GO:0009617 | response to bacterium | 7 | 566 | 0.000 |
| GO:0051345 | positive regulation of hydrolase activity | 7 | 652 | 0.000 |
| GO:0051223 | regulation of protein transport | 7 | 661 | 0.000 |
| GO:0007155 | cell adhesion | 7 | 705 | 0.000 |
| GO:1903530 | regulation of secretion by cell | 7 | 733 | 0.000 |
| GO:0022008 | neurogenesis | 7 | 1650 | 0.001 |
| GO:0009891 | positive regulation of biosynthetic process | 7 | 1847 | 0.002 |
| GO:2001141 | regulation of RNA biosynthetic process | 7 | 2976 | 0.020 |
| GO:0048513 | animal organ development | 7 | 3012 | 0.021 |
| GO:2000112 | regulation of cellular macromolecule biosynthetic process | 7 | 3324 | 0.034 |
| GO:0048247 | lymphocyte chemotaxis | 6 | 40 | 0.000 |
| GO:0030593 | neutrophil chemotaxis | 6 | 58 | 0.000 |
| GO:0002690 | positive regulation of leukocyte chemotaxis | 6 | 93 | 0.000 |
| GO:0070555 | response to interleukin-1 | 6 | 114 | 0.000 |
| GO:0045807 | positive regulation of endocytosis | 6 | 157 | 0.000 |
| GO:1903708 | positive regulation of hemopoiesis | 6 | 199 | 0.000 |
| GO:0051251 | positive regulation of lymphocyte activation | 6 | 253 | 0.000 |
| GO:0032496 | response to lipopolysaccharide | 6 | 298 | 0.000 |
| GO:0022407 | regulation of cell-cell adhesion | 6 | 360 | 0.000 |
| GO:0050708 | regulation of protein secretion | 6 | 439 | 0.000 |
| GO:1901701 | cellular response to oxygen-containing compound | 6 | 870 | 0.000 |
| GO:0051093 | negative regulation of developmental process | 6 | 979 | 0.001 |
| GO:0042592 | homeostatic process | 6 | 1494 | 0.004 |
| GO:0048699 | generation of neurons | 6 | 1538 | 0.004 |
| GO:0010557 | positive regulation of macromolecule biosynthetic process | 6 | 1717 | 0.007 |
| GO:0031328 | positive regulation of cellular biosynthetic process | 6 | 1807 | 0.008 |
| GO:0045123 | cellular extravasation | 5 | 33 | 0.000 |
| GO:2000107 | negative regulation of leukocyte apoptotic process | 5 | 61 | 0.000 |
| GO:0070098 | chemokine-mediated signaling pathway | 5 | 67 | 0.000 |
| GO:0071347 | cellular response to interleukin-1 | 5 | 90 | 0.000 |
| GO:0071346 | cellular response to interferon-gamma | 5 | 97 | 0.000 |
| GO:0051897 | positive regulation of protein kinase B signaling | 5 | 106 | 0.000 |
| GO:0032680 | regulation of tumor necrosis factor production | 5 | 136 | 0.000 |
| GO:1902107 | positive regulation of leukocyte differentiation | 5 | 162 | 0.000 |
| GO:0050707 | regulation of cytokine secretion | 5 | 181 | 0.000 |
| GO:0050731 | positive regulation of peptidyl-tyrosine phosphorylation | 5 | 184 | 0.000 |
| GO:0022409 | positive regulation of cell-cell adhesion | 5 | 227 | 0.000 |
| GO:0050878 | regulation of body fluid levels | 5 | 317 | 0.000 |
| GO:0002521 | leukocyte differentiation | 5 | 340 | 0.000 |
| GO:0043547 | positive regulation of GTPase activity | 5 | 353 | 0.000 |
| GO:0051090 | regulation of DNA-binding transcription factor activity | 5 | 373 | 0.000 |
| GO:0051222 | positive regulation of protein transport | 5 | 410 | 0.000 |
| GO:0045321 | leukocyte activation | 5 | 464 | 0.000 |
| GO:0071396 | cellular response to lipid | 5 | 464 | 0.000 |
| GO:0009991 | response to extracellular stimulus | 5 | 493 | 0.000 |
| GO:0007186 | G protein-coupled receptor signaling pathway | 5 | 824 | 0.002 |
| GO:0042493 | response to drug | 5 | 926 | 0.003 |
| GO:0022603 | regulation of anatomical structure morphogenesis | 5 | 1009 | 0.004 |
| GO:0048878 | chemical homeostasis | 5 | 1002 | 0.004 |
| GO:0009968 | negative regulation of signal transduction | 5 | 1147 | 0.007 |
| GO:0035556 | intracellular signal transduction | 5 | 1274 | 0.010 |
| GO:0009719 | response to endogenous stimulus | 5 | 1289 | 0.011 |
| GO:0033554 | cellular response to stress | 5 | 1370 | 0.013 |
| GO:0048468 | cell development | 5 | 1579 | 0.022 |
| GO:0010628 | positive regulation of gene expression | 5 | 1811 | 0.036 |
| GO:0071622 | regulation of granulocyte chemotaxis | 4 | 54 | 0.000 |
| GO:0001959 | regulation of cytokine-mediated signaling pathway | 4 | 97 | 0.000 |
| GO:0045621 | positive regulation of lymphocyte differentiation | 4 | 103 | 0.000 |
| GO:0050729 | positive regulation of inflammatory response | 4 | 116 | 0.000 |
| GO:0071222 | cellular response to lipopolysaccharide | 4 | 152 | 0.000 |
| GO:0050870 | positive regulation of T cell activation | 4 | 180 | 0.000 |
| GO:1901654 | response to ketone | 4 | 193 | 0.000 |
| GO:0007584 | response to nutrient | 4 | 199 | 0.000 |
| GO:1901215 | negative regulation of neuron death | 4 | 228 | 0.000 |
| GO:0001818 | negative regulation of cytokine production | 4 | 229 | 0.000 |
| GO:0044419 | interspecies interaction between organisms | 4 | 237 | 0.000 |
| GO:0045765 | regulation of angiogenesis | 4 | 287 | 0.000 |
| GO:0032147 | activation of protein kinase activity | 4 | 290 | 0.001 |
| GO:0001101 | response to acid chemical | 4 | 334 | 0.001 |
| GO:0002697 | regulation of immune effector process | 4 | 335 | 0.001 |
| GO:0009611 | response to wounding | 4 | 394 | 0.001 |
| GO:0071363 | cellular response to growth factor stimulus | 4 | 437 | 0.002 |
| GO:1903532 | positive regulation of secretion by cell | 4 | 436 | 0.002 |
| GO:0046903 | secretion | 4 | 501 | 0.003 |
| GO:0001568 | blood vessel development | 4 | 504 | 0.003 |
| GO:0010035 | response to inorganic substance | 4 | 505 | 0.003 |
| GO:0007167 | enzyme linked receptor protein signaling pathway | 4 | 551 | 0.004 |
| GO:0055082 | cellular chemical homeostasis | 4 | 663 | 0.007 |
| GO:0120035 | regulation of plasma membrane bounded cell projection organization | 4 | 712 | 0.008 |
| GO:0045664 | regulation of neuron differentiation | 4 | 714 | 0.008 |
| GO:0006468 | protein phosphorylation | 4 | 793 | 0.012 |
| GO:0009725 | response to hormone | 4 | 829 | 0.013 |
| GO:0044087 | regulation of cellular component biogenesis | 4 | 863 | 0.015 |
| GO:0071495 | cellular response to endogenous stimulus | 4 | 997 | 0.023 |
| GO:0044092 | negative regulation of molecular function | 4 | 1078 | 0.029 |
| GO:2000669 | negative regulation of dendritic cell apoptotic process | 3 | 7 | 0.000 |
| GO:0010759 | positive regulation of macrophage chemotaxis | 3 | 20 | 0.000 |
| GO:0032689 | negative regulation of interferon-gamma production | 3 | 30 | 0.000 |
| GO:0002548 | monocyte chemotaxis | 3 | 31 | 0.000 |
| GO:0097028 | dendritic cell differentiation | 3 | 34 | 0.000 |
| GO:0043491 | protein kinase B signaling | 3 | 38 | 0.000 |
| GO:0001961 | positive regulation of cytokine-mediated signaling pathway | 3 | 40 | 0.000 |
| GO:0007159 | leukocyte cell-cell adhesion | 3 | 48 | 0.000 |
| GO:0045124 | regulation of bone resorption | 3 | 48 | 0.000 |
| GO:0050710 | negative regulation of cytokine secretion | 3 | 56 | 0.000 |
| GO:0048260 | positive regulation of receptor-mediated endocytosis | 3 | 59 | 0.000 |
| GO:0014068 | positive regulation of phosphatidylinositol 3-kinase signaling | 3 | 64 | 0.000 |
| GO:0031100 | animal organ regeneration | 3 | 77 | 0.000 |
| GO:0032760 | positive regulation of tumor necrosis factor production | 3 | 82 | 0.000 |
| GO:0033273 | response to vitamin | 3 | 82 | 0.000 |
| GO:0070542 | response to fatty acid | 3 | 90 | 0.000 |
| GO:0042102 | positive regulation of T cell proliferation | 3 | 98 | 0.000 |
| GO:2001237 | negative regulation of extrinsic apoptotic signaling pathway | 3 | 98 | 0.000 |
| GO:1901655 | cellular response to ketone | 3 | 99 | 0.000 |
| GO:0033138 | positive regulation of peptidyl-serine phosphorylation | 3 | 103 | 0.001 |
| GO:0048661 | positive regulation of smooth muscle cell proliferation | 3 | 105 | 0.001 |
| GO:0002573 | myeloid leukocyte differentiation | 3 | 113 | 0.001 |
| GO:0002262 | myeloid cell homeostasis | 3 | 118 | 0.001 |
| GO:0007596 | blood coagulation | 3 | 118 | 0.001 |
| GO:0032675 | regulation of interleukin-6 production | 3 | 128 | 0.001 |
| GO:0001764 | neuron migration | 3 | 139 | 0.001 |
| GO:0001816 | cytokine production | 3 | 142 | 0.001 |
| GO:0048469 | cell maturation | 3 | 158 | 0.001 |
| GO:0045766 | positive regulation of angiogenesis | 3 | 168 | 0.002 |
| GO:0071229 | cellular response to acid chemical | 3 | 189 | 0.002 |
| GO:0050679 | positive regulation of epithelial cell proliferation | 3 | 193 | 0.002 |
| GO:0002699 | positive regulation of immune effector process | 3 | 196 | 0.002 |
| GO:0009615 | response to virus | 3 | 221 | 0.003 |
| GO:0044403 | symbiont process | 3 | 220 | 0.003 |
| GO:0006816 | calcium ion transport | 3 | 228 | 0.003 |
| GO:0051091 | positive regulation of DNA-binding transcription factor activity | 3 | 230 | 0.003 |
| GO:0097305 | response to alcohol | 3 | 228 | 0.003 |
| GO:0031668 | cellular response to extracellular stimulus | 3 | 239 | 0.004 |
| GO:0050714 | positive regulation of protein secretion | 3 | 271 | 0.005 |
| GO:1903829 | positive regulation of cellular protein localization | 3 | 297 | 0.006 |
| GO:0001525 | angiogenesis | 3 | 303 | 0.007 |
| GO:0035690 | cellular response to drug | 3 | 325 | 0.008 |
| GO:0008015 | blood circulation | 3 | 376 | 0.011 |
| GO:0046649 | lymphocyte activation | 3 | 378 | 0.012 |
| GO:0032940 | secretion by cell | 3 | 384 | 0.012 |
| GO:0006874 | cellular calcium ion homeostasis | 3 | 388 | 0.012 |
| GO:0043254 | regulation of protein complex assembly | 3 | 409 | 0.014 |
| GO:0045666 | positive regulation of neuron differentiation | 3 | 429 | 0.016 |
| GO:0031346 | positive regulation of cell projection organization | 3 | 435 | 0.016 |
| GO:0009636 | response to toxic substance | 3 | 471 | 0.020 |
| GO:0071407 | cellular response to organic cyclic compound | 3 | 475 | 0.020 |
| GO:0044089 | positive regulation of cellular component biogenesis | 3 | 501 | 0.022 |
| GO:0000904 | cell morphogenesis involved in differentiation | 3 | 506 | 0.023 |
| GO:0090066 | regulation of anatomical structure size | 3 | 508 | 0.023 |
| GO:0032870 | cellular response to hormone stimulus | 3 | 513 | 0.023 |
| GO:0010975 | regulation of neuron projection development | 3 | 547 | 0.027 |
| GO:0040008 | regulation of growth | 3 | 686 | 0.047 |
| GO:0035684 | helper T cell extravasation | 2 | 2 | 0.000 |
| GO:0060754 | positive regulation of mast cell chemotaxis | 2 | 7 | 0.000 |
| GO:0048245 | eosinophil chemotaxis | 2 | 10 | 0.000 |
| GO:0032825 | positive regulation of natural killer cell differentiation | 2 | 12 | 0.000 |
| GO:0035457 | cellular response to interferon-alpha | 2 | 12 | 0.000 |
| GO:0045625 | regulation of T-helper 1 cell differentiation | 2 | 13 | 0.000 |
| GO:0032736 | positive regulation of interleukin-13 production | 2 | 14 | 0.000 |
| GO:0048246 | macrophage chemotaxis | 2 | 14 | 0.000 |
| GO:0002523 | leukocyte migration involved in inflammatory response | 2 | 16 | 0.001 |
| GO:0045779 | negative regulation of bone resorption | 2 | 16 | 0.001 |
| GO:0050901 | leukocyte tethering or rolling | 2 | 18 | 0.001 |
| GO:0030225 | macrophage differentiation | 2 | 19 | 0.001 |
| GO:0090026 | positive regulation of monocyte chemotaxis | 2 | 20 | 0.001 |
| GO:0045624 | positive regulation of T-helper cell differentiation | 2 | 22 | 0.001 |
| GO:0031295 | T cell costimulation | 2 | 24 | 0.001 |
| GO:0048010 | vascular endothelial growth factor receptor signaling pathway | 2 | 24 | 0.001 |
| GO:0002052 | positive regulation of neuroblast proliferation | 2 | 26 | 0.001 |
| GO:0034695 | response to prostaglandin E | 2 | 26 | 0.001 |
| GO:0070233 | negative regulation of T cell apoptotic process | 2 | 26 | 0.001 |
| GO:0002691 | regulation of cellular extravasation | 2 | 29 | 0.001 |
| GO:0043277 | apoptotic cell clearance | 2 | 29 | 0.001 |
| GO:0071354 | cellular response to interleukin-6 | 2 | 28 | 0.001 |
| GO:0002092 | positive regulation of receptor internalization | 2 | 30 | 0.002 |
| GO:0032733 | positive regulation of interleukin-10 production | 2 | 31 | 0.002 |
| GO:2000406 | positive regulation of T cell migration | 2 | 32 | 0.002 |
| GO:0032735 | positive regulation of interleukin-12 production | 2 | 35 | 0.002 |
| GO:0046718 | viral entry into host cell | 2 | 35 | 0.002 |
| GO:0050704 | regulation of interleukin-1 secretion | 2 | 36 | 0.002 |
| GO:0061844 | antimicrobial humoral immune response mediated by antimicrobial peptide | 2 | 36 | 0.002 |
| GO:0050918 | positive chemotaxis | 2 | 40 | 0.002 |
| GO:0010518 | positive regulation of phospholipase activity | 2 | 41 | 0.003 |
| GO:0001974 | blood vessel remodeling | 2 | 43 | 0.003 |
| GO:0001960 | negative regulation of cytokine-mediated signaling pathway | 2 | 46 | 0.003 |
| GO:0033209 | tumor necrosis factor-mediated signaling pathway | 2 | 49 | 0.003 |
| GO:0032720 | negative regulation of tumor necrosis factor production | 2 | 52 | 0.004 |
| GO:0042531 | positive regulation of tyrosine phosphorylation of STAT protein | 2 | 52 | 0.004 |
| GO:0007157 | heterophilic cell-cell adhesion via plasma membrane cell adhesion molecules | 2 | 53 | 0.004 |
| GO:0030168 | platelet activation | 2 | 53 | 0.004 |
| GO:0061098 | positive regulation of protein tyrosine kinase activity | 2 | 53 | 0.004 |
| GO:0071398 | cellular response to fatty acid | 2 | 53 | 0.004 |
| GO:0090303 | positive regulation of wound healing | 2 | 53 | 0.004 |
| GO:0031670 | cellular response to nutrient | 2 | 55 | 0.004 |
| GO:0050766 | positive regulation of phagocytosis | 2 | 56 | 0.004 |
| GO:0032722 | positive regulation of chemokine production | 2 | 59 | 0.004 |
| GO:0071333 | cellular response to glucose stimulus | 2 | 73 | 0.006 |
| GO:0032755 | positive regulation of interleukin-6 production | 2 | 82 | 0.008 |
| GO:0001776 | leukocyte homeostasis | 2 | 83 | 0.008 |
| GO:0001938 | positive regulation of endothelial cell proliferation | 2 | 88 | 0.008 |
| GO:0043154 | negative regulation of cysteine-type endopeptidase activity involved in apoptotic process | 2 | 91 | 0.009 |
| GO:0045639 | positive regulation of myeloid cell differentiation | 2 | 95 | 0.010 |
| GO:0034101 | erythrocyte homeostasis | 2 | 96 | 0.010 |
| GO:0002824 | positive regulation of adaptive immune response based on somatic recombination of immune receptors built from immunoglobulin superfamily domains | 2 | 102 | 0.011 |
| GO:0035821 | modification of morphology or physiology of other organism | 2 | 102 | 0.011 |
| GO:0002274 | myeloid leukocyte activation | 2 | 114 | 0.013 |
| GO:0010811 | positive regulation of cell-substrate adhesion | 2 | 117 | 0.014 |
| GO:0046660 | female sex differentiation | 2 | 125 | 0.015 |
| GO:0050715 | positive regulation of cytokine secretion | 2 | 131 | 0.016 |
| GO:0051250 | negative regulation of lymphocyte activation | 2 | 139 | 0.018 |
| GO:0008360 | regulation of cell shape | 2 | 142 | 0.019 |
| GO:0090316 | positive regulation of intracellular protein transport | 2 | 144 | 0.019 |
| GO:0031589 | cell-substrate adhesion | 2 | 149 | 0.020 |
| GO:0043433 | negative regulation of DNA-binding transcription factor activity | 2 | 150 | 0.020 |
| GO:0032874 | positive regulation of stress-activated MAPK cascade | 2 | 157 | 0.022 |
| GO:0022408 | negative regulation of cell-cell adhesion | 2 | 168 | 0.024 |
| GO:0043524 | negative regulation of neuron apoptotic process | 2 | 168 | 0.024 |
| GO:0000165 | MAPK cascade | 2 | 170 | 0.025 |
| GO:0070588 | calcium ion transmembrane transport | 2 | 175 | 0.026 |
| GO:0007411 | axon guidance | 2 | 180 | 0.027 |
| GO:0030198 | extracellular matrix organization | 2 | 180 | 0.027 |
| GO:0003018 | vascular process in circulatory system | 2 | 190 | 0.030 |
| GO:0043903 | regulation of symbiosis, encompassing mutualism through parasitism | 2 | 192 | 0.030 |
| GO:0000302 | response to reactive oxygen species | 2 | 200 | 0.033 |
| GO:0043901 | negative regulation of multi-organism process | 2 | 203 | 0.033 |
| GO:2000027 | regulation of animal organ morphogenesis | 2 | 203 | 0.033 |
| GO:0008202 | steroid metabolic process | 2 | 206 | 0.034 |
| GO:0031334 | positive regulation of protein complex assembly | 2 | 226 | 0.040 |
| GO:0043406 | positive regulation of MAP kinase activity | 2 | 232 | 0.041 |
| GO:0042110 | T cell activation | 2 | 244 | 0.045 |

1. KEGG signaling pathway enrichment analysis of differentially expressed protein

| Table S12 KEGG signaling pathway enrichment analysis of differentially expressed protein | | | | |
| --- | --- | --- | --- | --- |
| term ID | Term Description | Gene  Counts | Background  Gene Count | *P* |
| mmu04060 | Cytokine-cytokine receptor interaction | 11 | 252 | 0.000 |
| mmu04062 | Chemokine signaling pathway | 5 | 179 | 0.000 |
| mmu04668 | TNF signaling pathway | 4 | 108 | 0.000 |
| mmu01521 | EGFR tyrosine kinase inhibitor resistance | 3 | 79 | 0.000 |
| mmu04657 | IL-17 signaling pathway | 3 | 91 | 0.000 |
| mmu04151 | PI3K-Akt signaling pathway | 3 | 349 | 0.011 |
| mmu04620 | Toll-like receptor signaling pathway | 2 | 98 | 0.011 |
| mmu04510 | Focal adhesion | 2 | 195 | 0.026 |
| mmu04630 | Jak-STAT signaling pathway | 2 | 161 | 0.023 |
| mmu04514 | Cell adhesion molecules (CAMs) | 2 | 158 | 0.023 |

1. Viability of RBL-2H3 cells.

| Table S13 Viability of RBL-2H3 cells | | |
| --- | --- | --- |
| group | mean | SEM |
| blank | 100.00% | 1.49% |
| TMDCD 20 μg/ml | 91.88% | 0.69% |
| TMDCD 40 μg/ml | 80.25% | 0.78% |
| TMDCD 80 μg/ml | 74.29% | 1.63% |

1. The release of β-hexosaminidase in RBL-2H3 cells.

| Table S14 The release of β-hexosaminidase in RBL-2H3 cells | | |
| --- | --- | --- |
| group | mean | SEM |
| blank | 14.71 | 0.38531 |
| model | 61.68 | 0.34133 |
| dexamethasone | 51.70 | 0.47388 |
| TMDCD 20 μg/ml | 57.46 | 0.26277 |
| TMDCD 40 μg/ml | 57.69 | 0.36423 |
| TMDCD 80 μg/ml | 55.57 | 0.68673 |

1. The release of histamine in RBL-2H3 cells.

| Table S15 The release of histamine in RBL-2H3 cells (pg/ml) | | |
| --- | --- | --- |
| group | mean | SEM |
| blank | 318.445 | 13.07586 |
| model | 567.3725 | 34.50367 |
| dexamethasone | 407.4625 | 1.78742 |
| TMDCD 20 μg/ml | 479.785 | 17.39777 |
| TMDCD 40 μg/ml | 463.495 | 32.98183 |
| TMDCD 80 μg/ml | 442.8425 | 24.74304 |

1. Traditional Chinese medicine in TMDCD


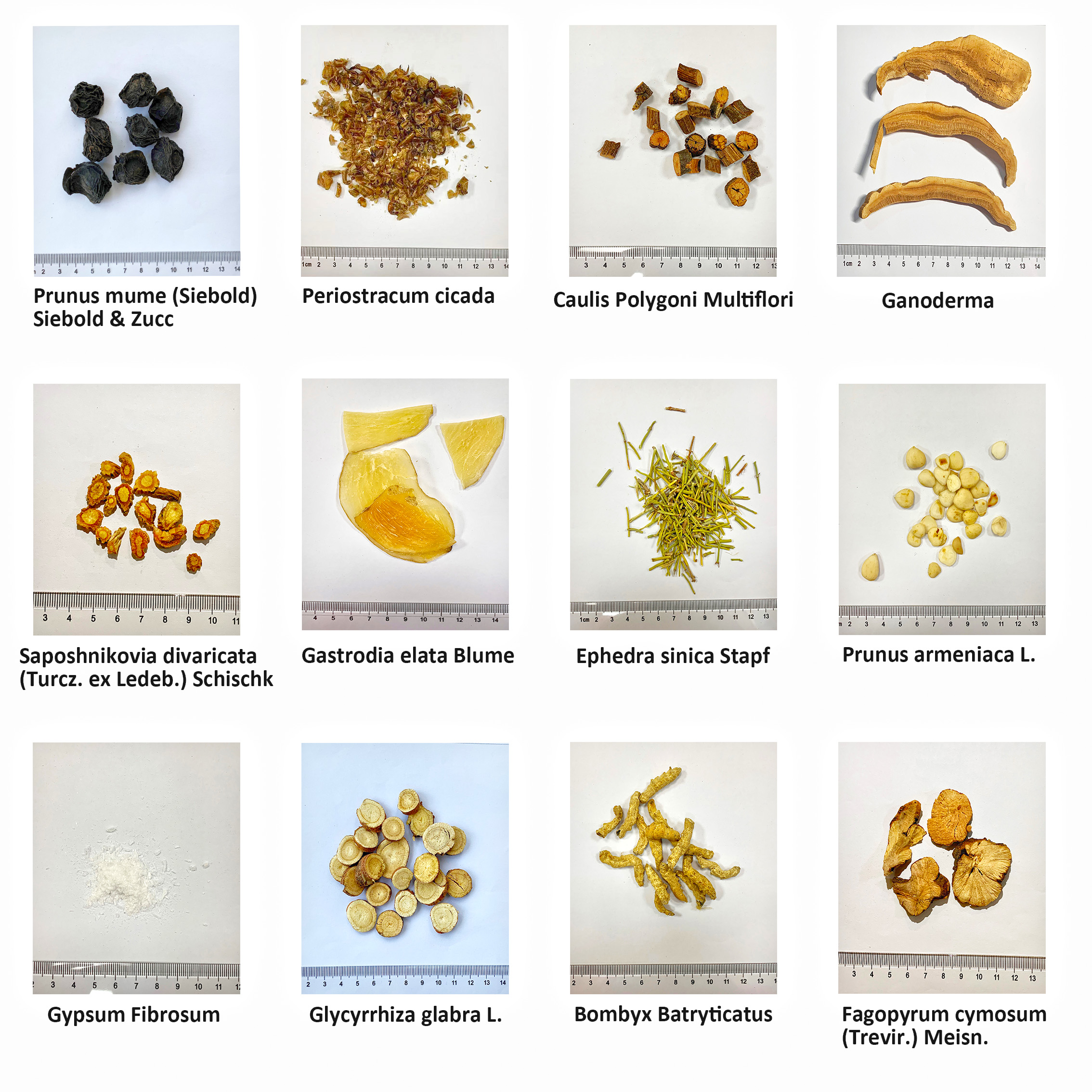


Fig. S1 Traditional Chinese medicine in TMDCD

1. LC-MS positive and negative ion chromatogra
2.
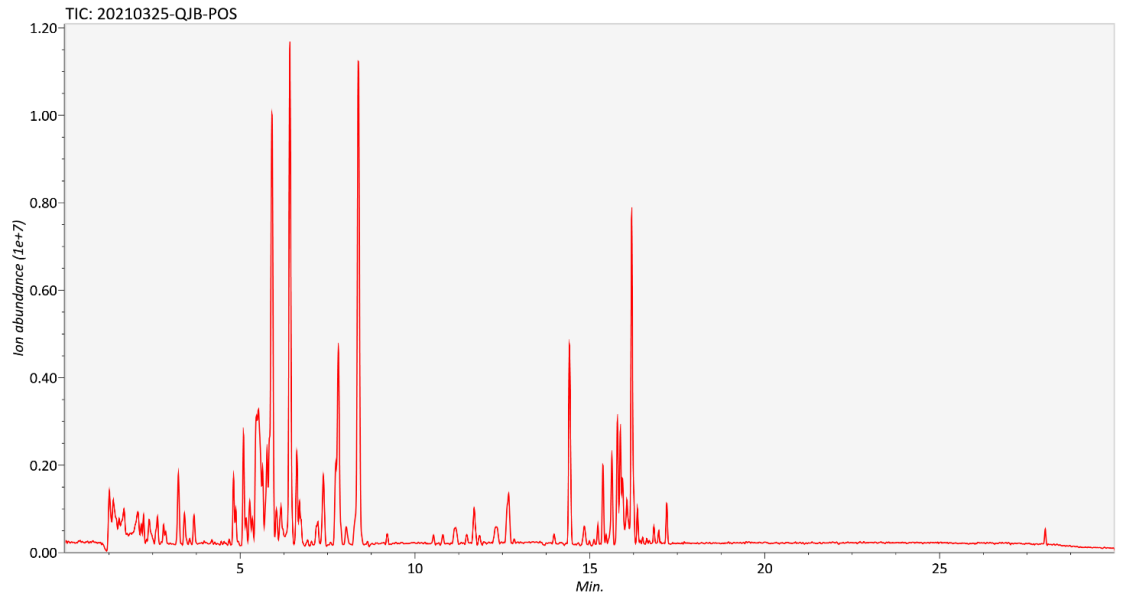

3.
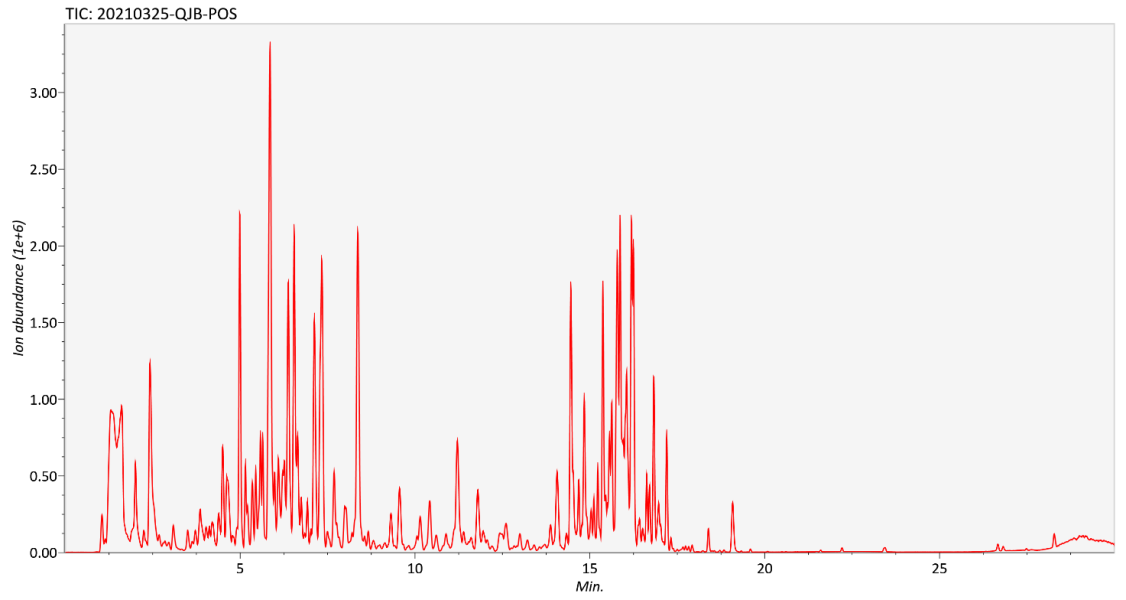


Fig. S2 LC-MS positive and negative ion chromatogram. (A) Positive ion chromatogram, (B) Negative ion chromatogram

1. Diff staining of inflammatory cells in BALF

a
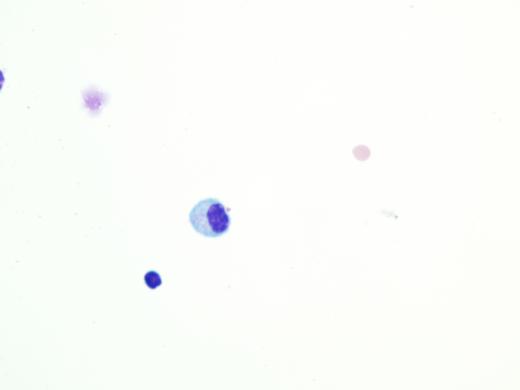
 b
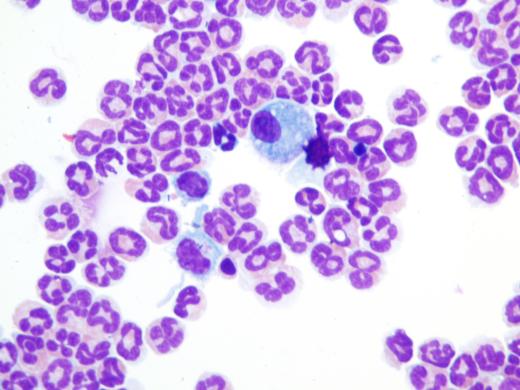


c
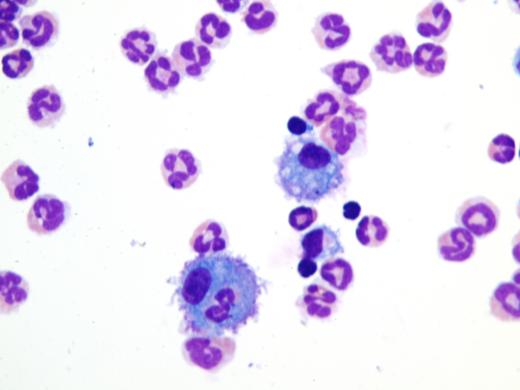
 d
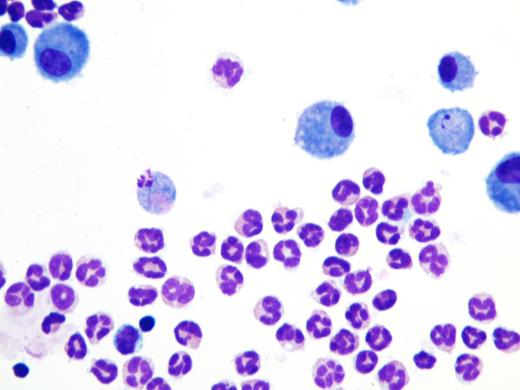


Fig. S3 Diff staining of inflammatory cells in BALF. a. NC group, b. OVA group, c. OVA+Dex, d. OVA+TMDCD

raw data: https://www.jianguoyun.com/c/sd/1320430/6a76b96b66a48d7f
